# Molecular Probing of the Microscopic Pressure at Contact Interfaces

**DOI:** 10.1021/jacs.4c01312

**Published:** 2024-05-02

**Authors:** Chao-Chun Hsu, Allen Chu-Hsiang Hsu, Chun-Yen Lin, Ken-Tsung Wong, Daniel Bonn, Albert M. Brouwer

**Affiliations:** † van ’t Hoff Institute for Molecular Sciences, University of Amsterdam, Science Park 904, 1098 XH Amsterdam, The Netherlands; ‡ Department of Chemistry, National Taiwan University, and Institute of Atomic and Molecular Science, Academia Sinica, Taipei 10617, Taiwan; § Van der Waals-Zeeman Institute, Institute of Physics, University of Amsterdam, Science Park 904, 1098 XH Amsterdam, The Netherlands

## Abstract

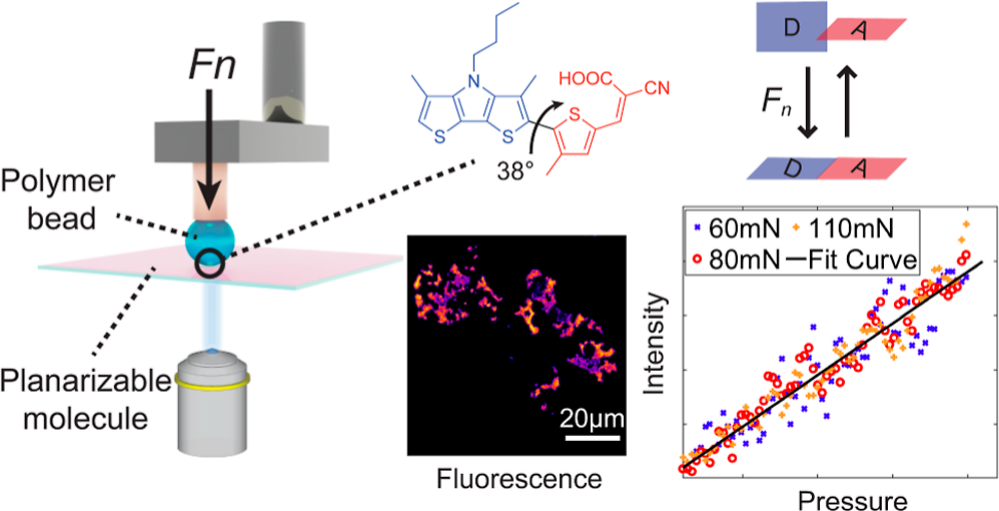

Obtaining insights into friction at the nanoscopic level and being able to translate these into macroscopic friction behavior in real-world systems is of paramount importance in many contexts, ranging from transportation to high-precision technology and seismology. Since friction is controlled by the local pressure at the contact it is important to be able to detect both the real contact area and the nanoscopic local pressure distribution simultaneously. In this paper, we present a method that uses planarizable molecular probes in combination with fluorescence microscopy to achieve this goal. These probes, inherently twisted in their ground states, undergo planarization under the influence of pressure, leading to bathochromic and hyperchromic shifts of their UV–vis absorption band. This allows us to map the local pressure in mechanical contact from fluorescence by exciting the emission in the long-wavelength region of the absorption band. We demonstrate a linear relationship between fluorescence intensity and (simulated) pressure at the submicron scale. This relationship enables us to experimentally depict the pressure distribution in multiasperity contacts. The method presented here offers a new way of bridging friction studies of the nanoscale model systems and practical situations for which surface roughness plays a crucial role.

## Introduction

When two objects come into contact, friction—the tangential force—acts to resist relative motion between them. While friction can lead to complications, such as wear, machinery malfunctions, and seismic events, it is also essential for everyday human locomotion. A deeper understanding of frictional interfaces, in relatively complex geometries such as rough surfaces in contact, can greatly influence the design of everyday systems.

According to the classical laws of Amontons and Coulomb, sliding motion sets in when the shear stress at the interface exceeds the product of the friction coefficient and the normal pressure.^1,2^ This is a simple rule for macroscopic contact, but at a rough interface, both the shear and the normal stress may vary widely within the contact area.^3−5^ Current tribology studies either attempt to consider multiasperity systems at a macroscopic level trying to find an effective description of the roughness or zoom in on single asperities on the nanoscale.^6−10^ Often, however, parameters such as the coefficient of friction (CoF), pressure, and adhesion display apparent inconsistencies when comparing nano- and macroscales,^11,12^ making it unclear how nanoscale friction measurements can be used to infer something about macroscopic friction. To be able to describe how a frictional contact is broken at the onset of sliding and to bridge scales, it is important to measure both the microscopic attributes (e.g., local shear stress, local pressure) and the microscopic contact area in multiasperity systems.^13^

It remains challenging to image the real contact area (*A*_r_) in multiasperity contacts with microscopic resolution, let alone simultaneously acquire local information about the stresses or pressure. Svetlizky and Fineberg and Scheibert et al. employed reflection microscopy to examine real contact areas, leveraging the refractive index difference at the contact interface.^6,7,14^ Pau et al. and Wiertlewski et al. used ultrasonic waves in the surface to measure the real contact area in hard and soft materials, respectively.^15,16^ In previous work, we used a fluorescent molecule which in its excited state can report on the local free volume or nanoviscosity.^17,18^ This probe is nearly planar in its ground state but will twist after excitation, which opens a nonradiative decay pathway that quenches the fluorescence.^19^ Upon confinement by decreasing the free space around the molecule, the geometry change is blocked for the excited state, resulting in a strong fluorescence enhancement. We exploited this photophysical property by introducing the probes into a contact interface where the contact provides the confinement, allowing us to visualize the real contact area and the nanoviscosity with submicron resolution.^18,20^ Despite these advancements, there is still a notable lack of methods that can provide direct, local-scale force measurements critical for understanding friction.

Here, we present a methodology to image the local normal pressure using a dye that responds to pressure via shifts of absorption and fluorescence spectra. Fluorescence techniques allowing to directly probe local forces using mechanochromism are widely applied in the studies of polymer systems.^21^ Color changes under the influence of force can originate from several physical effects, ranging from changes in intermolecular interactions in crystals and aggregates to bond breaking (mechanochemistry)^22−24^ and more gradual conformational changes that affect molecular color. Notably, most of these studies examine the tension stresses in bulk, typically by chemically cross-linking the probe within the bulk material. In recent works, methods were presented to visualize and quantify the microscopic shear stress using a mechanofluorochromic dye based on the ground-state isomerization of spirolactam probes.^25,26^

In contrast to “binary” molecular switching, gradual force-induced structural changes can be revealed by studying the absorption and fluorescence spectra. Matile et al. used a molecular push–pull system in which the probe features a rotatable bond capable of planarization in an ordered medium.^27^ This probe, when embedded in a lipid membrane, can detect membrane tension during cell fission through chalcogen-bonding.^28−30^ In investigations of polymer fracture, Saito et al. employed flapping molecules. Initially V-shaped, these molecules can flatten when compressed in a polymer matrix, resulting in molecular planarization and a bathochromic shift in the fluorescence. By correlating this color change with ratiometric mapping, local pressure within the polymer matrix can be visualized.^31−34^ More recently, Sommer et al. incorporated a twisted donor–acceptor molecule in a polymer matrix to study the stress–strain behavior of the polymer.^100^ By using the constrained geometries simulate external force molecular simulation method,^35^ they observed that the dihedral angle between the donor and acceptor is reduced under stress, leading to a bathochromic shift in both absorption and emission spectra that can be used as a stress indicator.^36^ However, limited research has been conducted on molecular behavior under static compression, which may differ significantly from hydrostatic pressure where forces are evenly applied.^36,37^

**Figure 1:**
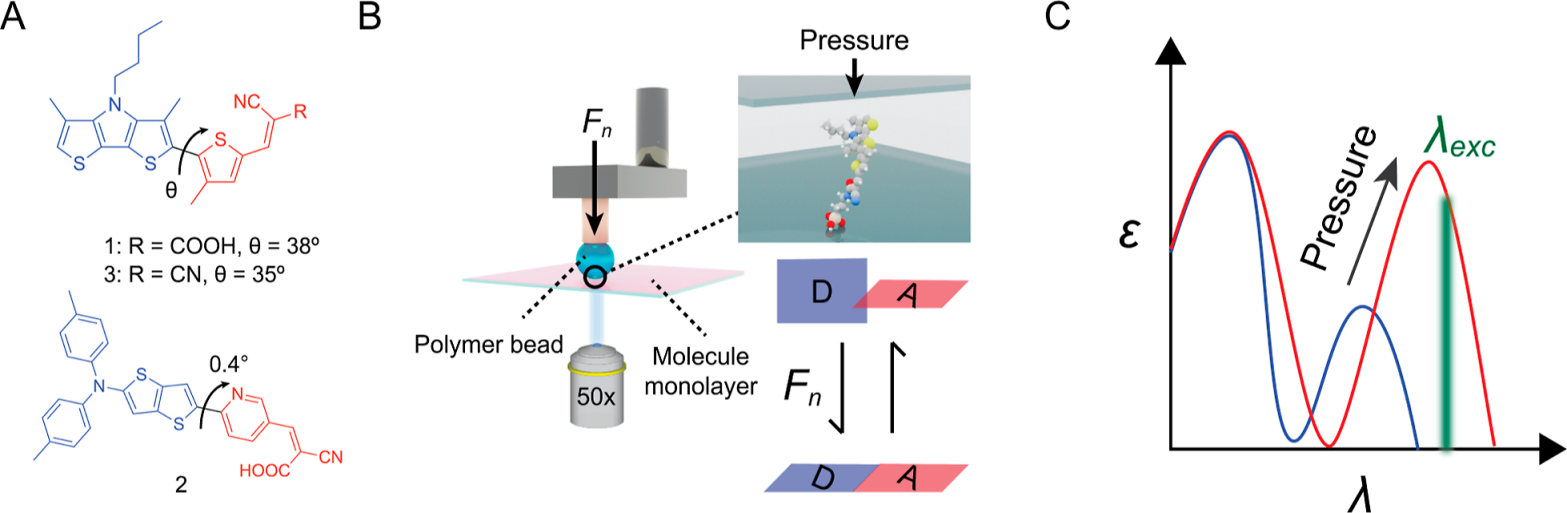
(A) Structures of probes **1**, **2**, and **3**, including dihedral angles θ between donor and acceptor units as calculated using PBE0/CC-pVTZ. (B) Schematic illustration of the concept of a planarizable probe to image surface contact pressure: a polymer bead pushes onto a surface-linked molecular layer with normal force *F*_n_, reversibly decreasing the internal dihedral angle between donor (indicated as D) and acceptor (indicated as A). (C) Illustration of absorption spectra showing the bathochromic shift and the increase in the molar absorption coefficient ε under increasing static pressure. To probe the absorption change, the emission is recorded with excitation at wavelength λ_exc_.

Here, we use fluorescence microscopy with a newly designed surface-immobilized probe (molecule **1** in Figure 1A) to map both the real contact area and the local pressure in a mechanical contact between a rough polymer bead and a smooth glass surface. As illustrated in Figure 1B, an external force leads to a reduced dihedral angle between the electron donating and accepting parts of the chromophore. As a result, the absorption band redshifts and becomes stronger, which can be probed in fluorescence imaging by exciting at the red edge of the absorption band (Figure 1C). Thus, the fluorescence intensity image will contain information about the local contact pressure at the interface. For comparison, we study molecule **2** (Figure 1A), which is similar to **1** but not twisted, and compound **3**, which is much more soluble than **1** and suitable for studying the effect of hydrostatic pressure in solution.

## Results and Discussion

**Scheme 1:**
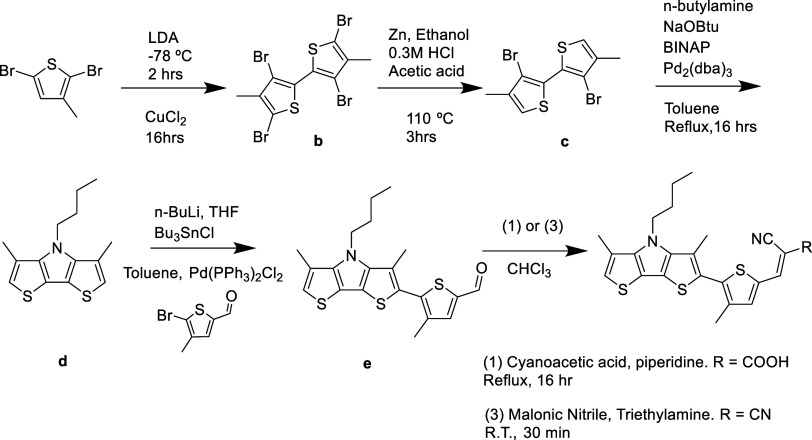
Synthetic Pathway and Reaction Conditions Used to Obtain Compounds **1** and **3**

The synthesis of the donor building block of compounds **1** and **3** was achieved via a modification of the methods developed by Suga et al. and Goto et al.^38−40^ We linked the donor and acceptor by Stille coupling, followed by a Knoevenagel condensation to obtain pure **1** and **3** (Scheme 1). The detailed synthetic steps can be found in the Supporting Information.

**Figure 2:**
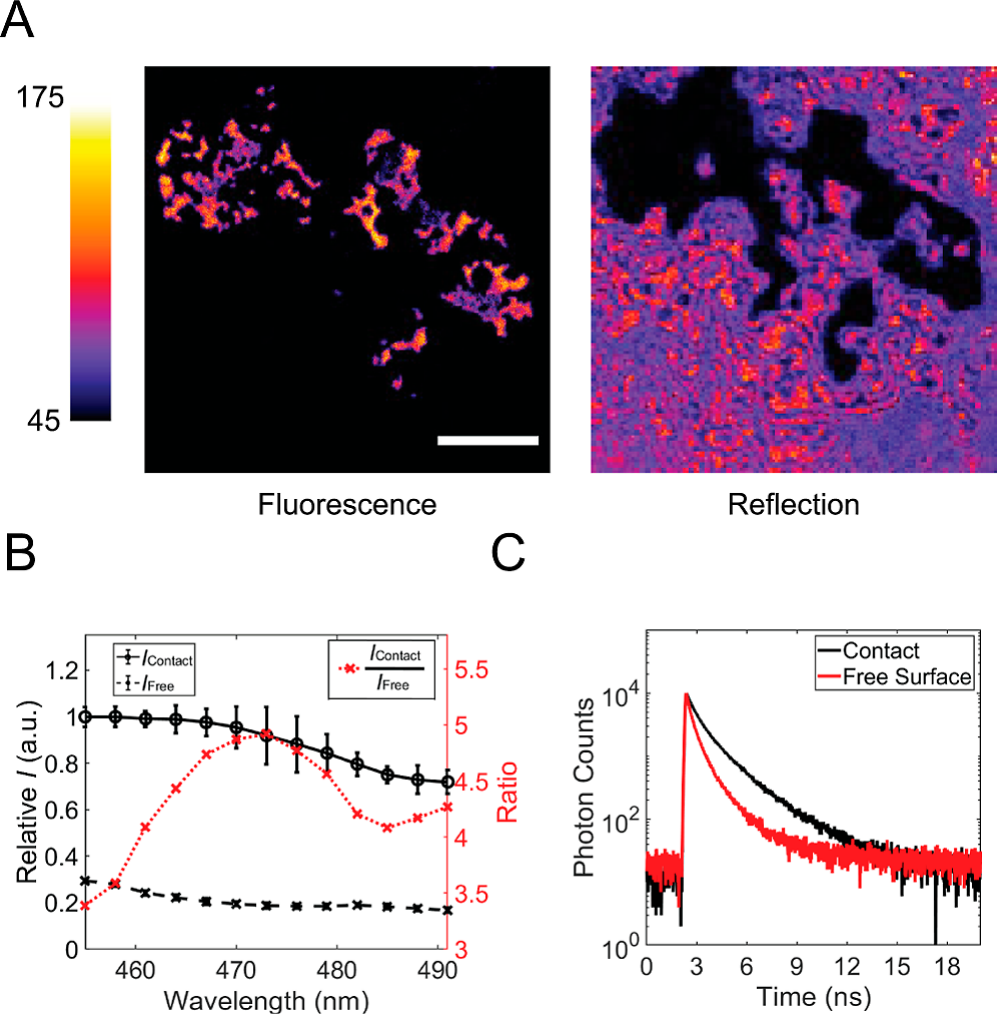
Contact experiment using surface-immobilized molecule **1**. (A) Contact imaging in fluorescence and reflection modes. The topography of the polymer bead is shown in Figure S3. Scale bar: 20 μm. Color scale bar indicates photon counts. (B) Excitation spectra of the probe layer with and without contact. (C) Time-correlated single-photon counting for the probe with and without contact. Fitted average lifetimes: 1.50 ns (contact) and 1.09 ns (no contact).

We performed contact experiments to examine the pressure sensitivity of molecule **1** under static contact. To this end, the probe was chemically anchored on an amino-functionalized glass surface using amide bonds (see Scheme S2).^25^ The experimental setup combined a rheometer arm, to which a poly(methyl methacrylate) (PMMA) bead was attached eccentrically, with an inverted fluorescence lifetime confocal microscope.^18^ By descending the polymer bead using a precision stage, we created contacts with controlled normal forces, which were measured by the rheometer. As presented in Figure 2A, a typical measurement included capturing contact images in both reflection and fluorescence mode. In the fluorescence mode, the nonlubricating solvent DMSO (*n* = 1.479) was used to match the refractive indices at the interfaces.^17^ Similar results could be obtained with decalin (*n* = 1.481).

The reflection image revealed a diminished intensity in the contact area, owing to the higher reflectance at the air–glass interface compared to the PMMA–glass interface. Notably, we observed light interference fringes outside the primary contact region, reinforcing previous findings that thresholding methods are unsuitable for determining the contact area in this imaging mode.^41^ In contrast, fluorescence images, taken with 488 nm excitation, enabled us to delineate the contact area using the measured local fluorescence intensity. This approach offers enhanced detail, allowing for the accurate identification of the real contact area using the unbiased Otsu threshold method.^18,42^ On the other hand, no contrast (Figure S2) was observed when using molecule **2** as the pressure probe (λ_ex_ = 560 nm). This confirms the necessity of a twisted donor–acceptor structure to achieve a pressure-dependent spectrum. Detailed descriptions of the experiments can be found in the Supporting Information.

To investigate the reason for the probe’s augmented fluorescence intensity within the contact area, we determined the photophysical properties of the molecule at both the contact interface and at the free surface. Partial excitation spectra were obtained by quantifying the average emitted photon counts while varying the excitation wavelength between 455 and 491 nm. Emission spectra were acquired using the spectrometer integrated in our microscope (see Supporting Information). The excitation spectra, depicted in Figure 2B, reveal a notable increase in contact, with a maximum enhancement of ∼5× at 475 nm. As we will show below, this increase results mostly from a pronounced enhancement in the conjugation of the molecules due to planarization of the molecules, which leads to a red shift of the absorption band and an increase in the oscillator strength.^27,28^ The emission spectra show little difference between the emissive species at the free surface and the contact zone as demonstrated in Figure S4. This is expected because the locally excited states in push–pull π-electron systems tend to have a smaller twist angle than the ground state. As demonstrated in Table S1, we used TDDFT to calculate the excited state geometries of molecules **1** and **3**, and the dihedral angles in the excited state were found to be 16.8 and 17.4°, respectively. Thus, there will be little or no pressure effect on the excited-state geometry and, thereby, on the fluorescence spectrum. The fluorescence lifetime, on the other hand, is almost 50% higher within the contact region than at the free surface (1.50 vs 1.09 ns). This can be explained by the suppression of nonradiative decay in the more rigid environment,^43^ which gives a corresponding increase in the fluorescence quantum yield. Yet, the difference in contrast observed in Figure 2B surpasses 400%. Therefore, the contrast primarily stems from the planarization of the molecule in the ground state, which shifts the absorption onset to the red and amplifies its electronic absorbance at the red edge of the absorption spectrum, in line with the schematic representation in Figure 1C.

**Figure 3:**
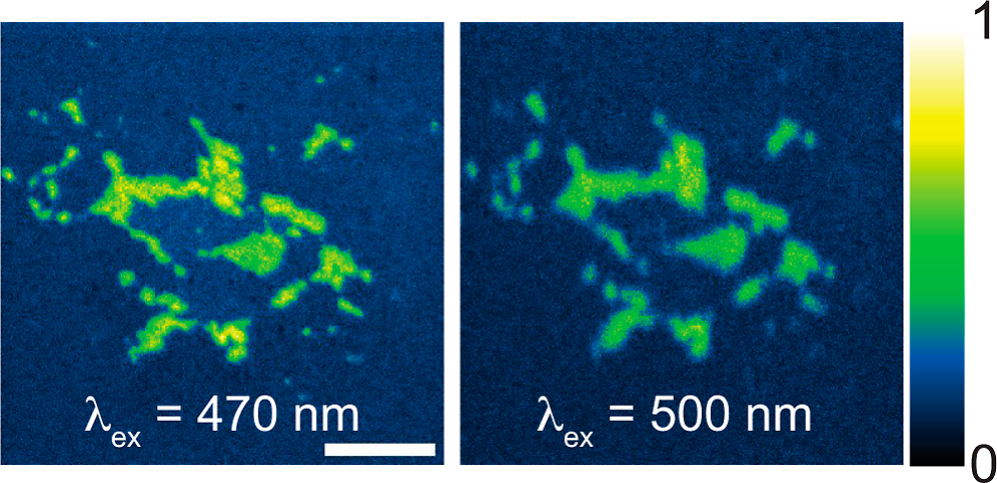
Contact images taken at 470 and 500 nm, with intensity rescaled to be between 0 and 1. Scale bar: 20 μm.

The intensity of the fluorescence depends on the excitation wavelength due to the pressure dependence of the absorption spectrum. To determine the wavelength giving maximum contrast in the images, we performed contact imaging at different wavelengths. The contrast—represented as the ratio of intensities between the contact and noncontact region—displays a clear correlation with the excitation wavelength with a maximum <*I*>_contact_/<*I*>_free_ = 5 at 470 nm, which matches with the maximum enhancement in the excitation spectra (Figure 2B). In Figure 3, we show the same contact imaged using excitation at 470 nm and at 500 nm, subsequently normalizing the intensity values to be between 0 and 1. The clarity of the foreground in the image excited at 470 nm is markedly superior to that excited at 500 nm. Through this contrast analysis, we determined that the optimal wavelength for pressure visualization is at 470 nm.

**Figure 4:**
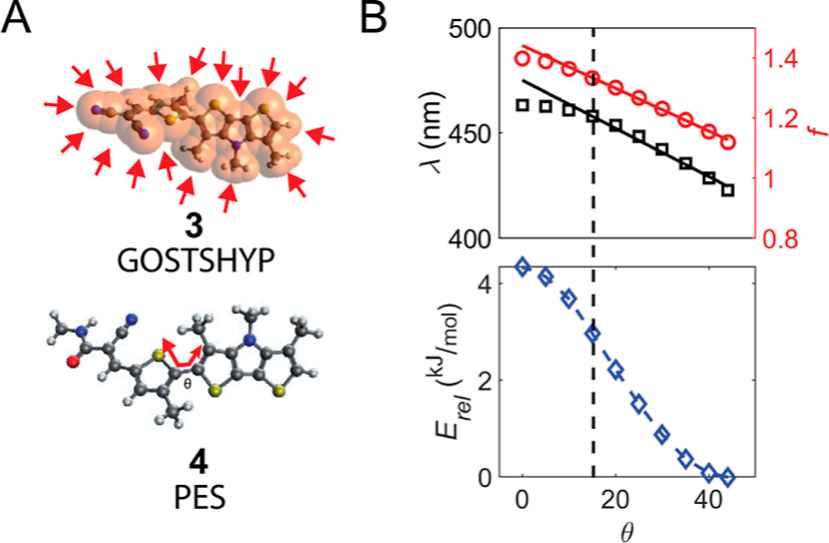
Quantum chemical calculations. (A) Illustration of the geometry optimization under hydrostatic pressure of molecule **3** and illustration of the dihedral angle θ in molecule **4**. (B) Potential energy scan, showing the energy (blue), oscillator strength (*f*, red), and absorption wavelength λ (black) vs dihedral angle of molecule **4**. Solid lines are linear fits. The vertical dashed line corresponds to the optimized dihedral angle in the S_1_ state.

**Figure 5:**
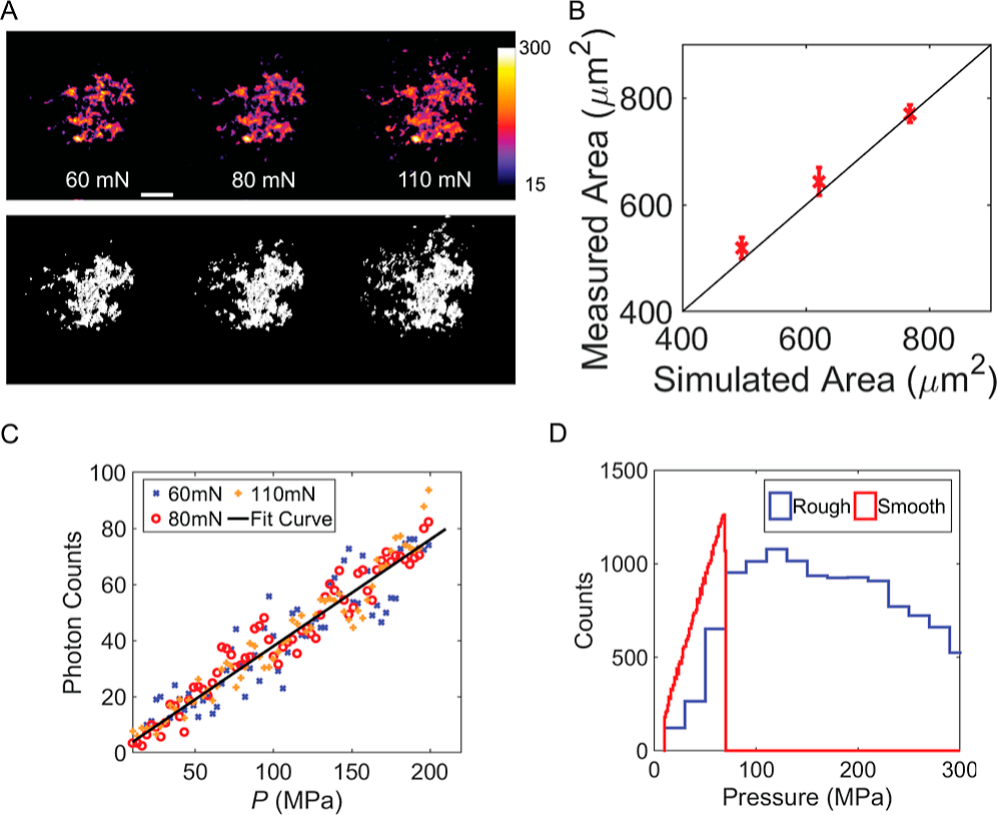
(A) Local pressure mapping under varying normal forces obtained from the fluorescence data (top) and the contact area simulations (bottom) under different applied normal forces. Polymer bead parameters and topography detailed in the Supporting Information (Figure S6, Table S2). (B) Comparison of contact areas derived from fluorescence imaging and simulations. (C) Correlation between simulated pressure and measured fluorescence via pixel-to-pixel calibration at different applied normal forces. The black solid line is a linear fit. (D) Pressure distributions over the contact areas of a rough polymer bead (experimental, blue line) and an ideal smooth bead (simulated, red line).

The excitation spectra of the immobilized probe with and without contact (Figure 2B) were obtained in a small wavelength range, limited by the tunability of the excitation light source. To obtain a picture of pressure-induced spectral changes over a more complete wavelength range, we determined the excitation and emission spectra of reference compound **3** in toluene solution under hydrostatic pressure. Although the spectra of **3** are red-shifted, the trends of excitation energy and oscillator strengths as a function of the dihedral angle are the same as for **1** (Figure S9). Since the compounds studied in this work show solvatochromic shifts in absorption and emission (see ESI, Figure S8), a nonpolar solvent is preferred for this experiment because its polarity does not change much with pressure.^44−46^ However, compound **1** is not sufficiently soluble in toluene, and therefore reference compound **3** was used for this experiment. As shown in Figure S8C (Supporting Information), the expected changes in the excitation spectrum were observed, but the enhancement in excitation under hydrostatic pressure was much smaller (<2.5× at 270 MPa) than that of immobilized **1**, as shown in Figure 2B. To investigate the reasons for this difference, we performed DFT calculations, as shown in Figure 4. To model the effect of hydrostatic pressure, we performed geometry optimizations using the Gaussians on surface tesserae simulate hydrostatic pressure method.^47^ The dihedral angle was θ = 42.8° without added pressure and θ = 40.6° at 1000 MPa (see Supporting Information for more details), which is a much higher pressure than the hydrostatic pressures explored here (≤270 MPa, Figure S8) or the contact pressures (≤300 MPa, see Figure 5). This result suggests that isotropic pressure has less effect than the directional pressure in the contact to effectively planarize the molecules. To investigate the energetics and the spectral response of the probes when squeezed toward planarity under anisotropic force, we performed potential energy scans. We determined the energy (*E*), oscillator strength (*f*), and excitation wavelength (λ_exc_) as a function of the dihedral angle of the donor and acceptor building blocks between 65 and 0°.^48^ In addition to compounds **1** and **3**, we also included compound **4**, in which the carboxylic acid group of **1** is replaced with an *N*-methyl amide to mimic the linker to the surface (Figure 4A). Figure 4B shows the results for the amide molecule **4**. Comparison of the results from all molecules (Figure S9) reveals that molecule **4** has a larger dihedral angle at the energy minimum than molecules **1** and **3**. The more strongly electron-withdrawing acid and cyano groups shift the minimum to somewhat smaller dihedral angles and the absorption spectrum to the red. The amide in solution has a larger dihedral angle and consequently undergoes a larger geometry change upon planarization, resulting in the large bathochromic shift and absorption enhancement at the contact interface.

According to our contact experiment, immobilized molecule **1** gives essentially the same fluorescence spectra with and without contact. The calculated dihedral angle in the S_1_ energy minimum is close to 15° for all three compounds and both the S_1_ energy and the emission wavelengths barely change when decreasing the dihedral angle to ∼0°. In the ground state, the energy surfaces along the inter-ring twisting coordinate are very shallow. Thus, planarization is easy to achieve using an externally applied anisotropic force. The predicted excitation spectrum of **4** exhibits a redshift of >30 nm, while the oscillator strength increases by >25% upon planarization. The net result, before reaching the dihedral angle at the S_1_ energy minimum, is an approximately linear increase (solid lines in Figure 4B) in the absorption cross section with pressure at the red edge of the absorption band.

Next, we evaluated whether the probe could capture the local pressure and the real contact area simultaneously, within a single measurement. We recorded the contact images of a randomly rough polymer bead (different from the one used in the experiments shown in Figures 2 and 3) while increasing the normal force from 60 to 110 mN in steps. Before initiating the contact experiment, the topography of the polymer bead was measured using an optical profilometer (Figure S6) exactly at the area where it was going to touch the countersurface. Based on these data, we performed a boundary element method (BEM) calculation using TriboSolver to obtain the simulated contact area and the pressure profile.^49^ The input parameters can be found in Table S2. As shown in Figure 5A, the contact patterns from experiment and simulation are in good quantitative agreement. With increasing normal force, the contact area grows, reflecting the elastoplasticity of the material.^4^Figure 5B depicts the contact areas derived from both methodologies, emphasizing their congruence, and highlighting the minimal variation between them. This indicates that the fluorescence method, utilizing molecular planarization, can be used for an accurate representation of the real contact area.

To probe the local pressure via the fluorescence method, we correlated pixel-by-pixel the BEM calculated pressures with the measured photon counts. Following background subtraction, the experimental and simulated images were aligned using a feature-matching algorithm, a built-in function in MATLAB. Pixels were binned based on local pressure increments of 3 MPa, and the corresponding fluorescence intensities in those assigned pixels were averaged. The relationship between local intensity and local pressure, observed across a range of normal forces, shows a consistent linear trend (Figure 5C), which likely emanates from the increasing average degree of planarization with increasing pressure.

In previous studies, we used fluorescent molecular rotors to determine the real contact area *A*_r_.^18,50^ For the rotors, the suppression of nonradiative decay pathways due to restrained molecular mobility in contact areas makes the fluorescence intensity higher there, allowing us to see whether the surfaces touch. Although the pressure should influence the local mobility gradually, the rotor probe responds in an almost binary on/off fashion to contact.^18,50,51^ As a result, physical parameters such as stress and pressure are averaged as *F*_n_/*A*_r_ over the full contact area, resulting in the loss of local information. In contrast, the methodology described here reveals detailed local information, enabling us to quantify the pressure distribution in multiasperity contacts. In Figure 5D, we present a histogram of the pressure distribution within the real contact area of the polymer bead pushing down onto a probe layer with an applied normal force of 110 mN. We compare this with the simulated pressure distribution of a smooth surface under the same applied force (full 2D pressure map in Figure S7), representing a single asperity contact. Notably, a marked difference can be observed between the two pressure distributions: the pressure of a rough surface ranges from 0 to 300 MPa, while for the smooth surface, this range is from 0 to 70 MPa; this, of course, happens since in the rough case, fewer asperities carry the load. The differences suggest that contacts exhibit diverse frictional behaviors, for instance, in the preslipping regime. Preslipping is believed to be locally initiated at points where the ratio of tangential to normal force surpasses a threshold value, represented by the CoF.^25,52,53^ When localized preslipping takes place, the “slipping” contact gets smaller, leading to the remaining contact area bearing an increased normal force.^6,50^ Increasing the normal load per contact area, therefore, requires greater tangential load locally to break the contact due to a nonlinear relationship between diminishing contact area and tangential load.^6^ Therefore, the initial local pressure distribution and the application of lateral forces over time critically influence the evolution of the contact area and, thus, the critical shear stress to initiate the slipping in the presliding stage. This leads to observable differences, like roughness-dependent friction coefficients, macroscopically.^13^ Previous experiments have reported conflicting findings: while some discovered that rougher surfaces exhibited a higher CoF, others found them to be more slippery.^5,54−56^ However, a comprehensive and systematic investigation is needed to reconcile these disparate observations, i.e., different material, roughness, and tangential application methods. Through our proposed methodology, we envision a potential pathway to bridge frictional phenomena across both nanoscopic and macroscopic scales.

## Conclusions and Future Perspectives

In this work, we have introduced a new method to simultaneously visualize the microscopic real contact area and the local pressure using fluorescence microscopy. We used a probe molecule that possesses a twisted ground state and undergoes progressive planarization when exposed to an increasing static contact pressure. This ground-state geometrical change results in a bathochromic shift of the excitation spectra, accompanied by an enhancement in absorbance. Fluorescence microscopy allows us to quantitatively evaluate the contact area by indirectly probing the absorption change at a carefully selected wavelength. A key aspect of the design is the low energy barrier along the twisting coordinate, that can be overcome by relatively small forces. Both calculations and experiments show a difference between the effect of isotropic hydrostatic pressure and the sensitivity to directional pressure at the interface; the latter induces a larger extent of planarization. Comparing the contact images at varying normal forces against simulated counterparts reveals the precision of the fluorescence method in measuring real contact areas. By implementing pixel-to-pixel comparison, we have established that the measured local intensity exhibits a linear dependence on local pressure. Consequently, when comparing the experimental pressure distribution of a rough polymer bead with a perfectly smooth surface, we found that the rough contact exhibits a broader pressure distribution, peaking at a value significantly higher than its smooth counterpart. The observed variations suggest that the evolution of contact area, the local shear force needed to break contact, and the resultant macroscopic CoF are heavily influenced by the initial pressure distribution and the application of tangential load. The key factors affecting the macroscopic CoF remain a debated topic in tribology. Investigating the roughness-dependent CoF alongside pressure distribution mapping could provide further insights. Additionally, examining how the contact area reduces over time with different materials and time-dependent tangential loads could be informative. The methodology proposed in this study offers a promising approach to unify our understanding of friction at nanoscopic and macroscopic levels.

## Data Availability

Additional data, including the content of the figures, are available at 10.21942/uva.25102280.

## Supporting Information Available

Materials and methods, synthetic procedure of planar molecule **2**, surface functionalization procedure of **1** and **2**, additional figures referred to in the text, spectroscopic characterization of the molecules, summary of (TD)DFT calculations, and parameters for BEM simulation (PDF)
Atomic coordinates of molecule **3** optimized with and without hydrostatic pressure (TXT)
