# Supplementary material for: Molecular Probing of the Microscopic Pressure at Contact Interfaces
Source: J Am Chem Soc. 2024 May 2;146(19):13258–65. doi: 10.1021/jacs.4c01312 (PMC11099955; doi:10.1021/jacs.4c01312)
Supplement: Supplementary file 1 — ja4c01312_si_001.pdf [file ja4c01312_si_001.pdf]

Molecular Probing of the Microscopic Pressure at Contact Interfaces

Chao-Chun Hsu[1*], Allen Chu-Hsiang Hsu[2], Chun-Yen Lin[2], Ken-Tsung Wong[2], Daniel Bonn[3], Albert M. Brouwer[1*]

[1] van 't Hoff Institute for Molecular Sciences, University of Amsterdam, Science Park 904, 1098 XH Amsterdam, The Netherlands

[2] Department of Chemistry, National Taiwan University, and Institute of Atomic and Molecular Science Academia Sinica Taipei 10617, Taiwan

[3] van der Waals-Zeeman Institute, Institute of Physics, University of Amsterdam, Science Park 904, 1098 XH Amsterdam, The Netherlands

Email: c.c.hsu@uva.nl (C.C. Hsu) a.m.brouwer@uva.nl (A.M. Brouwer)

Table of Contents

Materials and Methods **4**
Synthesis of Molecule 1 and 3 .... **4**
Synthesis of 3,3',5,5'-tetrabromo-4,4'-dimethyl-2,2'-bithiophene (b) 4
Synthesis of 3,3'-dibromo-4,4'-dimethyl-2,2'-bithiophene (c) 4
Synthesis of 4-butyl-3,5-dimethyl-4H-dithieno[3,2-b:2',3'-d]pyrrole (d) 4
Synthesis of 5-(4-butyl-3,5-dimethyl-4H-dithieno[3,2-b:2',3'-d]pyrrol-2-yl)-4-methylthiophene-2-carbaldehyde (e) 4
Synthesis of 2-((5-(4-butyl-3,5-dimethyl-4H-dithieno[3,2-b:2',3'-d]pyrrol-2-yl)-4-methylthiophen-2-yl)methyl-ene)malononitrile, molecule 3 5
Synthesis of 3-(5-(4-butyl-3,5-dimethyl-4H-dithieno[3,2-b:2',3'-d]pyrrol-2-yl)-4-methylthiophen-2-yl)-2-cyanoacrylic acid, molecule 1 5
Synthesis of molecule 2 .... **5**
Scheme S1. Synthetic procedures and conditions to obtain molecule **2**. 6
Synthesis of N, N-di-p-tolylthieno[3,2-b]thiophen-2-amine 6
Synthesis of 6-(5-(di-p-tolylamino)thieno[3,2-b]thiophen-2-yl)nicotinaldehyde 6
Synthesis of 2-cyano-3-(6-(5-(di-p-tolylamino)thieno[3,2-b]thiophen-2-yl)pyridin-3-yl)acrylic acid, molecule 2 6
Surface Immobilization **7**
Scheme S2. 7
Contact Images and Photophysical Properties **7**
Quantum Chemical calculations **9**
Scheme S3. 9
Table S1. 9
Supplementary Figures and Tables **10**
Figure S1. 10
Figure S2. 10
Figure S3. 10
Figure S4 11
Figure S5 11
Table S2. 11
Figure S6. 12
Figure S7. 12
Figure S8 13

Figure S9. ... 14

NMR, Infrared, and Mass Spectra ... **15**

Figure S10 ^{1}H NMR spectrum of compound **c**. Solvent: $CDCl_3$. ... 15

Figure S11 ^{1}H NMR spectrum of compound **d.** Solvent: $CDCl_3$ ... 16

Figure S12 ^{1}H NMR spectrum of compound **e**. Solvent: $CDCl_3$. ... 17

Figure S13 ^{1}H NMR spectrum of molecule **3**. Solvent: acetone-d_6. ... 18

Figure S14 ^{13}C NMR spectrum of molecule **3**. Solvent: acetone-d_6. ... 19

Figure S15 ^{1}H NMR spectrum of molecule **1**. Solvent: dmso-d_6. ... 20

Figure S16 ^{13}C NMR spectrum of molecule **1**. Solvent: dmso-d_6 ... 21

Figure S17 ... 22

Figure S18 ... 22

Figure S19 Infrared spectrum of compound **1** (solid powder). ... 23

Figure S20 ^{1}H NMR spectrum of 6-(5-(di-p-tolylamino)thieno[3,2-b]thiophen-2-yl)nicotinaldehyde. Solvent: CD_2Cl_2 ... 24

Figure S21 ^{13}C NMR spectrum of 6-(5-(di-p-tolylamino)thieno[3,2-b]thiophen-2-yl)nicotinaldehyde. Solvent: CD_2Cl_2 ... 25

Figure S22 ^{1}H NMR spectrum of molecule **2**. Solvent: CD_2Cl_2. ... 26

Figure S23 ^{13}C NMR spectrum of molecule **2**. Solvent: CD_2Cl_2. ... 27

Figure S24 Mass spectrum of compound **2**. ... 28

References ... **29**

Materials and Methods

Chemicals and solvents for syntheses, purification, and analyses were purchased from Sigma-Aldrich unless otherwise specified. The acceptor building block 5-bromo-4-methylthiophene-2-carbaldehyde was acquired from Fluorochem Ireland, while 2,5-dibromo-3-methylthiophene was purchased from Abcr GmbH. With the exception of ethanol, solvents were dried using 4Å molecular sieves (Carl Roth GmbH) before reactions. NMR spectra were recorded using a Bruker AV II 400 spectrometer, and subsequently analyzed with MestReNova. The infrared spectrum of molecule **1** in Figure S19 was taken using a PerkinElmer Spectrum™ 3 spectrometer.

Synthesis of Molecule 1 and 3

The synthesis of the new probe **1** was achieved by the method modified from the previous studies developed by Suga et al. and Goto et al.[1,2] In the first step, dibromo-methylthiophene was reacted with lithium disopropylamide (LDA) to generate Li-Br exchange.[3] The intermediate was directly transferred to a dry reaction flask with $CuCl_2$ under argon at -78 ˚C, whereupon it formed compound **b**. The compound was reduced by Zn powder; in the final step, a nucleophilic substitution of amine to bromine was performed to afford compound **d**. Next, we coupled the thiophene with **d** with Stille coupling. In the final step, the molecule was modified with a Knoevenagel condensation to afford a dicyano group or a cyanoacetic acid group. Detailed synthetic steps are described below.

Synthesis of 3,3',5,5'-tetrabromo-4,4'-dimethyl-2,2'-bithiophene (b)

In a reaction flask (A) diisopropylamine (4.77 mL, 34.0 mmol) and THF (60 mL) were mixed under Ar atmosphere. After stirring at -78 ˚C for 30 minutes to achieve a steady temperature, 1.6 M n-BuLi (19.5 mL) was added dropwise. The solution was stirred for an additional hour to produce fresh LDA. Subsequently, 2,5-dibromo-3-methylthiophene (7.27 g, 28.3 mmol) was introduced dropwise over ~15 minutes. The reaction was maintained at -78 ˚C for 2 hrs. In another reaction flask (B), $CuCl_2$ (2.69 g, 20 mmol) was placed under Ar atmosphere at -78 ˚C, and the mixture from (A) is directly transferred under Ar. Stirring continued for 16 hrs, after which the reaction mixture was quenched with 1M HCl and extracted with $CHCl_3$. The reaction product was purified using flash chromatography (Eluent = hexane) to afford compound b (5.77g, 80%).

Synthesis of 3,3'-dibromo-4,4'-dimethyl-2,2'-bithiophene (c)

Molecule b (5 g, 9.80 mmol) was combined with ethanol (30 mL), 0.3M HCl (6.1 mL), and acetic acid (6.1 mL) in a reaction flask. The mixture was brought to reflux (80 ˚C). Zinc dust (1.8 g, 27.5 mmol) was introduced in 5 portions over 30 mins to the mixture. After the zinc dust addition, the reaction mixture was refluxed for an additional 3 hrs. After cooling, the mixture was neutralized by 0.1M NaOH and extracted with $CHCl_3$. The organic phase was treated with $MgSO_4$ to remove water and dried under vacuo. The crude product was precipitated in 5 mL of methanol at 0 ˚C under vigorously stirring to obtain pure molecule **c** (1.8 g, 52%). ^{1}H NMR (400 MHz, $CDCl_3$) δ 7.16 (q, J = 1.1 Hz, 1H), 2.31 (d, J = 1.0 Hz, 3H).

Synthesis of 4-butyl-3,5-dimethyl-4H-dithieno[3,2-b:2',3'-d]pyrrole (d)

Compound c (1 g, 2.84 mmol), NaOtBu (660 mg, 6.87 mmol), $Pd_2(dba)_3$ (260 mg, 0.28 mmol), and BINAP (177 mg, 0.28 mmol) were combined in dry toluene (20 mL) under Ar atmosphere. The mixture was refluxed and n-butylamine (214 mg, 2.93 mmol) was added. After 16 hrs of reflux, the solution was extracted with EA and washed with several portions of water. The organic phase was dried with $MgSO_4$, and the solvent was evaporated in vacuo. The mixture was separated by chromatography (Eluent = 80:1 Hexane:EA) to obtain pure compound **d** (580 mg, 78%). ^{1}H NMR (400 MHz, $CDCl_3$) δ 6.69 (s, 2H), 4.43 – 4.34 (m, 2H), 2.49 (s, 6H), 1.80 (p, J = 7.7 Hz, 2H), 1.41 (h, J = 7.5 Hz, 3H), 0.96 (t, J = 7.4 Hz, 3H).

Synthesis of 5-(4-butyl-3,5-dimethyl-4H-dithieno[3,2-b:2',3'-d]pyrrol-2-yl)-4-methylthiophene-2-carbaldehyde (e)

Compound d (300 mg, 1.14 mmol) was dissolved in THF (20 mL), and the solution was cooled to -78 ˚C. n-Butyllithium (0.75 mL, 1.6M in n-hexane) was added dropwise. After an hour at room temperature, the mixture was

cooled to -78 ˚C again before adding tributyltin (390 mg, 1.2 mmol). The crude was gradually raised to room temperature in 5 hrs. The resulting mixture was washed with saturated potassium fluoride and extracted with ether to quench excess tin compound. The solution was reintroduced to the same reaction flask with 80 mg Bis(triphenylphosphine)palladium(II) dichloride (80 mg, 0.114 mmol), and 5-bromothiophene-2-carbaldehyde (234 mg, 1.14 mmol). After dissolving in 20 mL of toluene, the reaction mixture was refluxed for 16 hrs, cooled to ambient temperature, extracted with dichloromethane (DCM) and washed with several portions of water. The organic phase was dried with $MgSO_4$, and the solvent was evaporated in vacuo. The mixture was separated by chromatography (Eluent = 4:1 Hexane:DCM to pure DCM) to afford pure **e** (200 mg, 45%). ^1H NMR (400 MHz, $CDCl_3$) δ 9.88 (s, 1H), 7.64 (s, 1H), 6.79 (d, J = 1.4 Hz, 1H), 4.52 – 4.40 (m, 2H), 2.53 (d, J = 1.2 Hz, 3H), 2.45 (s, 3H), 2.30 (s, 3H), 1.85 (p, J = 7.8 Hz, 2H), 1.46 (h, J = 7.6 Hz, 2H), 1.01 (t, J = 7.3 Hz, 3H).

Synthesis of 2-((5-(4-butyl-3,5-dimethyl-4H-dithieno[3,2-b:2',3'-d]pyrrol-2-yl)-4-methylthiophen-2-yl)methylene)malononitrile, molecule 3

Compound e (50 mg, 0.129 mmol) and cyanoacetic acid (21 mg, 0.326 mmol) were dissolved in $CHCl_3$ (20 mL). Piperidine (1 mL) was added, and the mixture was kept at room temperature for 30 mins. The product was extracted with $CHCl_3$ and separated by chromatography (Eluent = ethylacetate) to obtain pure molecule **3** (13 mg, 23%). ^1H NMR (400 MHz, Acetone-d_6) δ 7.31 (s, 1H), 6.92 (d, J = 1.3 Hz, 1H), 6.24 (s, 2H), 4.60 – 4.52 (m, 2H), 2.56 (d, J = 1.1 Hz, 3H), 2.49 (s, 3H), 2.27 (s, 3H), 1.87 (dq, J = 11.7, 7.9, 6.2 Hz, 2H), 1.51 (dd, J = 15.1, 7.5 Hz, 2H), 1.00 (t, J = 7.4 Hz, 3H). ^{13}C NMR (101 MHz, Acetone) δ 159.18, 151.08, 143.34, 143.32, 136.96, 135.36, 133.37, 132.53, 125.04, 122.60, 120.28, 116.91, 116.33, 115.18, 115.06, 80.00, 45.28, 35.23, 19.63, 14.14, 13.97, 13.23, 12.68. m/z: [M]+ calcd for $C_{23}H_{21}N_3S_3$ 435.0898; Found 435.0895

Synthesis of 3-(5-(4-butyl-3,5-dimethyl-4H-dithieno[3,2-b:2',3'-d]pyrrol-2-yl)-4-methylthiophen-2-yl)-2-cyanoacrylic acid, molecule 1

In a reaction flask, compound e (50 mg, 0.129 mmol) and cyanoacetic acid (100 mg, 1.17 mmol) were dissolved in $CHCl_3$ (20 mL). The mixture was refluxed under Ar atmosphere and then treated with 1 mL of piperidine. After an 8-hour reaction, the solution was washed with 1M HCl and extracted with DCM. Purification via chromatography (Eluent = 15:1 to 8:1 DCM : MeOH) yielded pure compound **1** (39 mg, 67%). ^1H NMR (400 MHz, DMSO-d_6) δ 8.02 (s, 1H), 7.60 (s, 1H), 7.02 (d, J = 1.2 Hz, 1H), 4.45 (t, J = 7.9 Hz, 2H), 2.50 (s, 3H), 2.40 (s, 3H), 2.18 (s, 3H), 1.77 (p, J = 7.8 Hz, 2H), 1.39 (h, J = 7.4 Hz, 2H), 0.94 (t, J = 7.4 Hz, 3H). ^{13}C NMR (101 MHz, DMSO) δ 143.65, 143.33, 140.67, 138.02, 137.65, 136.85, 130.66, 130.49, 124.95, 122.87, 121.64, 120.45, 119.45, 118.96, 115.00, 114.93, 45.38, 35.34, 19.83, 14.97, 14.83, 14.13, 13.59. m/z: [M + H]+ calcd for $C_{23}H_{22}N_2O_2S_3$ 455.0922; Found 455.0906. IR spectrum see Figure S19.

Synthesis of molecule 2

To elucidate the planarization effect, we synthesized a model system utilizing the planar molecule **2**. The dihedral angle between the donor and acceptor moieties was 0.4˚ as determined by DFT calculation using the PBE0 hybrid functional and the CC-pVTZ basis set and the Polarizable Continuum Solvent model for toluene with the default settings in Gaussian16. Compound **2** is related to a series of compounds that have been employed in organic photovoltaics.[4,5] Details regarding the synthetic steps of the molecule can be found in Scheme S1 and the subsequent descriptions.

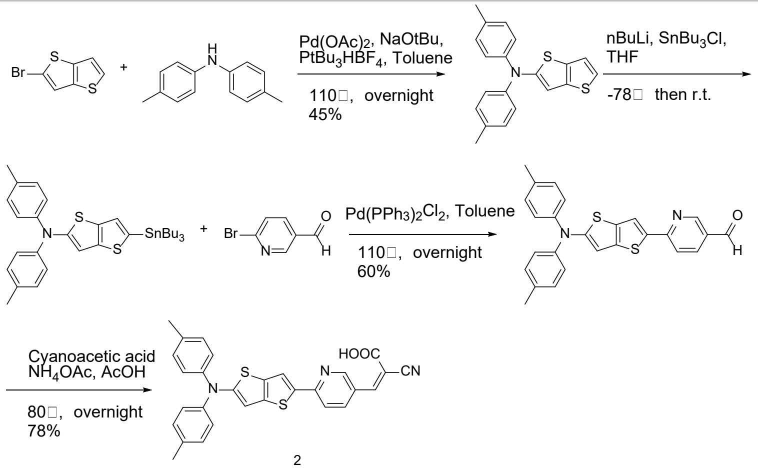

Scheme S1. Synthetic procedures and conditions to obtain molecule **2**.

Synthesis of N, N-di-p-tolylthieno[3,2-b]thiophen-2-amine

In a two-neck bottle, 2-bromothieno[3,2-b]thiophene (1.10 g, 5.02 mmol), di-*p*-tolylamine(1.09 g, 5.52 mmol), sodium *tert*-butoxide (0.96 g, 10.04 mmol), palladium(II) acetate (56.3 mg, 0.25 mmol) and tri-tert-butylphosphonium tetrafluoroborate (0.15 g, 0.5 mmol) were mixed under argon atmosphere. Toluene (25ml) was added, and the reaction mixture was heated to 110ºC for 16 hrs. Upon cooling, the mixture was diluted with dichloromethane and filtered. The solvent was evaporated, and the crude material was extracted with dichloromethane and washed successively with water and brine. The organic layer was collected and dried with $MgSO_4$. After evaporation of the solvent the product was purified using chromatography with the eluent EA/Hexane (1:10) and reprecipitation from DCM/MeOH to afford the title compound as a pale yellow solid (0.76 g, 45%). ^{1}H NMR (400 MHz, CD_2Cl_2) δ 7.33-7.30 (m, 1H), 7.17 (d, 4 Hz, 1H), 7.10-7.04 (m, 8H), 6.86 (s, 1H), 2.32 (s, 6H).

Synthesis of 6-(5-(di-p-tolylamino)thieno[3,2-b]thiophen-2-yl)nicotinaldehyde

To a stirred solution of *N*, *N*-di-p-tolylthieno[3,2-b]thiophen-2-amine (1.34 g, 4.00 mmol)in THF (40 mL) was added dropwise n-BuLi (1.6 M in hexane, 2.8 mL, 4.40 mmol) at -78ºC under argon and the reaction was continued for an additional 1 hr. Tributyltin chloride (1.3 mL, 4.60 mmol) was added to the solution at -78ºC, and it was brought back to ambient temperature. The mixture was extracted after 16 hrs with ether and washed with brine. The organic solution was dried over $MgSO_4$ and concentrated to give the reactant for the coupling reaction. The reactant was added to the reaction flash with 6-bromonicotinaldehyde (0.74 g, 4.00 mmol), and bis(triphenylphosphine) palladium(II) dichloride (0.28 g, 0.40 mmol). The reaction mixture was refluxed for 16 hrs in toluene (40 mL) under Ar atmosphere. After cooling to room temperature, the solvent was removed under reduced pressure, and the excess organotin reagent was removed by reprecipitation in pentane. The crude product was purified by column chromatography with DCM, and reprecipitation from DCM/MeOH to afford the title compound as a red solid (0.96 g, 60%). ^{1}H NMR (400 MHz, CD_2Cl_2) *δ* 9.95 (s, 1H), 8.79 (s, 1H), 8.02 (dd, *J* = 8, 2 Hz, 1H), 7.58 (d, *J* = 8.4 Hz 1H), 7.46(d, *J* = 4 Hz, 1H), 7.17-7.11 (m, 8H), 6.44 (d, *J* = 4.4 Hz, 1H), 2.34 (s, 6H) ^{13}C NMR (101 MHz, CD_2Cl_2) δ 189.8, 157.6, 157.4, 153.1, 144.7, 142.5, 141.22, 135.8, 134.1, 132.3, 130.0, 129.0, 123.9, 120.2, 118.0, 109.0, 20.9.

Synthesis of 2-cyano-3-(6-(5-(di-p-tolylamino)thieno[3,2-b]thiophen-2-yl)pyridin-3-yl)acrylic acid, molecule 2

6-(5-(di-p-tolylamino)thieno[3,2-b]thiophen-2-yl)nicotinaldehyde (0.9 g, 2.05 mmol), cyanoacetic acid (0.52 g, 6.15 mmol), and ammonium acetate (0.10 g, 1.23 mmol) were dissolved in acetic acid (40 ml), and the mixture was stirred at 80 ºC overnight. The solution was poured into water and the filtrate was collected. It was dissolved in $CHCl_3$ reprecipitation with pentane to afford the pour molecule **2**. 1H NMR (400 MHz, CD_2Cl_2) δ 8.56 (d, J = 9.2Hz, 1H), 8.23 (s, 1H), 7.77 (s, 1H), 7.71 (d, J = 9.2Hz), 7.68 (s, 1H), 7.13 (s, 8H), 6.65 (s, 1H), 2.33 (s, 6H), ^{13}C NMR 101 MHz, CD_2Cl_2) δ 206.44, 172.12, 160.57, 158.94, 152.82, 148.32, 142.36, 135.66, 133.92, 130.01, 129.86, 129.73, 124.64, 123.84, 123.24, 122.40, 106.86, 100.02, 91.45, 22.55. HRMS (m/z, MALDI, [M]+) Calcd. for $C_{29}H_{21}N_3O_2S_2$ 507.1070, found 507.1145.

Surface Immobilization

In Scheme S2, we show the surface functionalization procedure for **1** and **2** in a manner similar to our previous studies.[6,7] Clean coverslips were silanized by immersing them in a mixture of 2 mL of APTES, 80 mL of 96% EtOH, and 2 mL of 99% acetic acid, undergoing a 30 mins reaction at room temperature. The cover slips were rinsed with absolute ethanol and annealed in an oven at 1 bar, 130 °C for 24 hrs. Silanized coverslips were placed in a reaction flask with molecule **1** or **2** (20 mg, 0.044 or 0.035 mmol), 1-[bis(dimethylamino)-methylene]-1H-1,2,3-triazolo[4,5b]pyridinium 3-oxid hexafluorophosphate (HATU) (46 mg, 0.12 mmol), and N,N-diisopropylethylamine (DIPEA) (30 mg, 0.23 mmol). 65 mL of anhydrous dimethylformamide (DMF) was added. The immobilization was continued for 16 hours under argon at room temperature. The coverslips were cleaned with absolute ethanol with sonification to remove excess reagents and residual DMF after the immobilization. Surface homogeneity of the coverslip anchored with molecule **1** was tested by using confocal microscope as demonstrated in Figure S1.

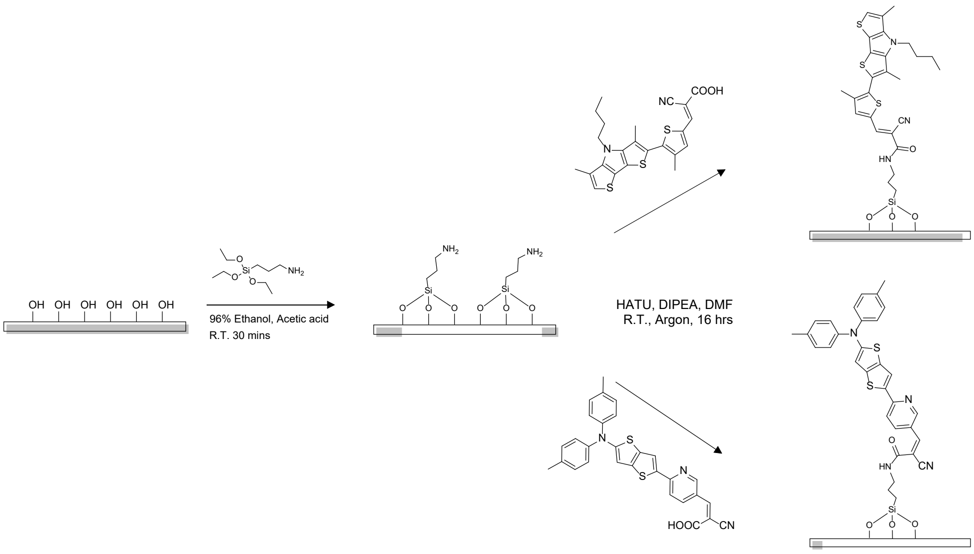

Scheme S2. Procedures for immobilizing molecules **1** and **2** on glass.

Contact Images and Photophysical Properties

Fluorescence lifetimes and fluorescence images were measured using a MicroTime 200 confocal microscope (PicoQuant GmbH) with an Olympus IX-71 microscope body and a 50 × 0.45 N.A. objective (SLMplan, Olympus). Various excitation wavelengths were generated with an NKT Supercontinuum Laser (SuperK Extreme Supercontinuum, NKT Photonics). Depending on the wavelength, different excitation filters were utilized: a FF409em filter (Semrock) for wavelengths between 455 and 476 nm and a bandpass (482/18, Semrock) for 479-491 nm to block unwanted light. A dichroic mirror (Z488RDC, Chroma) was used to reflect the excitation light, and the emitted/reflected light from the sample was filtered by the same dichroic mirror, a 488 nm notch filter (NF01-488U-25, Semrock), and a long pass filter (550FGL, Thorlabs). The light was then detected using time-resolved single photon counting (TCSPC) with a PDM Series detector (PicoQuant GmbH). Decay time traces were processed and fitted with SymPhoTime64 using the Tailfit method. Steady-state emission spectra were measured using an EMCCD camera (PhotonMAX, Princeton Instruments/Acton) attached to a spectrometer (Spectra Pro-150, Acton Research Instruments). The collected emission was filtered (496LP, Chroma) to exclude the excitation light. The wavelength of the spectrograph was calibrated with 3rd order polynomial fitting using the NKT Supercontinuum Laser at 500 nm, 530 nm, 560 nm, and 600 nm with a fixed power of 1.1 µW.

The contact experiment was realized by using a rheometer eccentrically mounted with a polymer bead (polystyrene (PS) or poly(methylmethacrylate)(PMMA)) on the scanning stage of the microscope. Reflective images were first captured on coverslips without probe molecules to identify the contact zone. Afterward, coverslips were switched to samples modified with either probe **1** or **2**, minimizing photobleaching under continuous intensive laser exposure. The pixel size was 229 nm/pixel. PMMA (1.45 mm diameter) and PS (1.55 mm diameter) polymer beads (Cospheric,

Somis California, US) were used without further modification. The contact image of probe **2** is presented in Figure S2, showing a negative contrast where brightness diminishes upon static contact.

The topographies of the polymer beads were recorded prior to the contact experiments using an optical profilometer (Keyence) with the pixel size set at 277 nm/pixel. Contact topographies are displayed in Figures S3 and S6. These topographies were used in the boundary element method (BEM) simulations for contact areas using an elastic-fully plastic model. The simulated images shown in Figure 5 were rescaled with a bilinear method to match the pixel size of the experimental images. Parameters were derived from earlier studies, with hardness measured by matching experimental and simulated contact areas as in Figure S5. Input parameters for simulations are in Table S2.

Emission spectra under contact are measured with the spectrometer on the confocal microscope as demonstrated in Figure S4. For excitation spectra and contrast analyses on the confocal microscope, a 50/50 beam splitter (Thorlabs) replaced the dichroic mirror. Fluorescence intensities at various excitation wavelengths were recorded using the image scanning mode with the scanning stage constantly moving in the contact zone or free surface to prevent possible photobleaching. The data was converted into time trace files using SymPhoTime64 with the intensity binned per second. Each wavelength had a fixed acquisition time of 10 seconds, i.e. 10 measurements for a single wavelength, for statistic validity. Throughout the measurements, the laser power was fixed at 1.1 μW to minimize the photobleaching, and the power was calibrated using a power meter (PM160, Thorlabs) prior to the experiment. All images and data were analyzed by using ImageJ and Matlab.[8]

To assess the probe's response under hydrostatic pressure, molecule **3** was dissolved in toluene and placed in a custom pressure cell, detailed in ref [6]. In summary, the device features a stainless-steel container with four sapphire windows. The sample solution, in a quartz cell with a movable Teflon piston cap, was positioned in a chamber filled with heptane. Pressure, up to ~3 kbar, was applied via a high-pressure screw piston pump. The device was integrated into a fluorescence spectrometer (SPEX Fluorolog 3-22 fluorimeter, Horiba), and emission was detected in right-angle mode. Spectral properties or molecule **3** under hydrostatic pressure are shown in Figure S8.

Quantum Chemical calculations

The ground state energy minima for the model probes in which the *n*-butyl groups of **1**, **2** and **4** were replaced by methyl groups (Scheme S3) were optimized using the PBE0 hybrid Density Functional with the CC-pVTZ basis set and the Polarizable Continuum Solvent model for toluene with the default settings in Gaussian16.[9] Potential energy scans along the twisting coordinate were performed by driving one of the S-C-C-C dihedral angles and optimizing all other coordinates as demonstrated in Figure 4B and Figure S9. At each point the absorption parameters were calculated using TD-DFT with the CAM-B3LYP functional. Excitation energies and oscillator strengths are shown in Figure S9. Values at the optimized geometries are reported in Table S1. Excited states were optimized at the CAM-B3LYP/CC-pVTZ level. The dihedral angles (average of the two S-C-C-C angles) are reported in Table S1. The fluorescence energies are calculated using the corrected linear response method.[10]

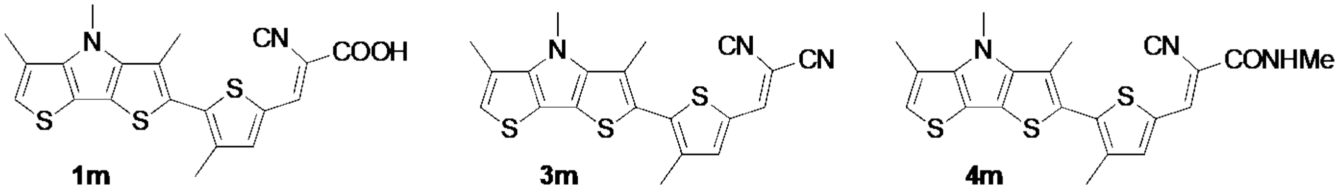

1m **3m** **4m**

Scheme S3. Structures of model compounds used in calculations.

Table S1. Results of (TD)DFT calculations

Compound	θ S_0 [a]	E_{exc} (eV)[b]	f[c]	θ S_1 [d]	E_{fluo} (eV) [e]
**3m**	34.7	2.67	1.17	17.4	2.42
**1m**	37.7	2.78	1.13	16.8	2.45
**4m**	42.1	2.93	1.12	15.5	2.49

[a] average of the S-C-C-C dihedral angles (PBE0/CC-pVTZ); [b] TDDFT excitation energy (CAM-B3LYP at PBE0/CC-pVTZ optimized S_0 geometry); [c] oscillator strength; [d] average of the two S-C-C-C dihedrals at the optimized geometry (CAM-B3LYP/CC-PVTZ); [e] fluorescence energy, calculated with corrected linear response[10].

We used the GOSTSHYP method (implemented in Q-Chem 6.1)[11] to calculate the geometry of molecule **3** as isolated molecule and under hydrostatic pressure.[12] In this case we did not use a solvent dielectric model, and the CC-PVDZ basis set. The default criteria for reaching a self-consistent field and for optimized geometry proved inadequate and they were tightened to SCF = 10^{-9} and gradient_convergence = 5×10^{-5}. The optimized geometries are provided as additional supporting information in xyz format in a plain text file.

Supplementary Figures and Tables

Figure S1 shows the fluorescence image of a coverslip on which molecule **1** was anchored. Figure S2 displays the fluorescence contact image with immobilized compound **2** as the probe, revealing a negative contrast characterized by decreased brightness upon contact formation.

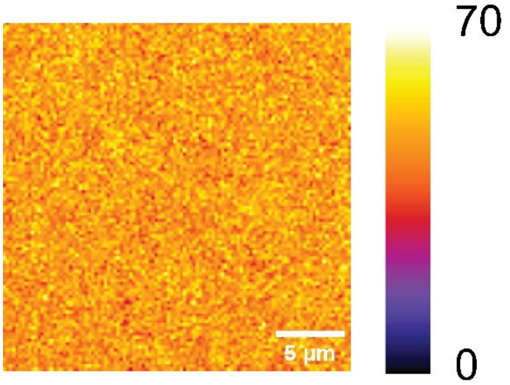

Figure S1. Fluorescence image of a coverslip with covalently linked compound **1** without contact application. λ_{ex} = 488 nm. The color scale bar represents photon counts.

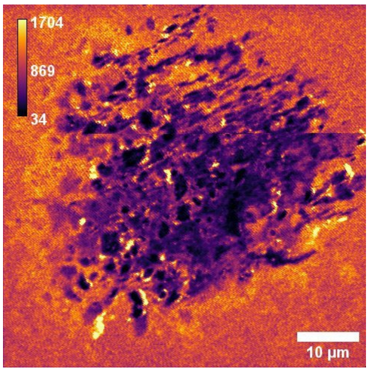

Figure S2. Contact image using molecule **2**, λ_{ex} = 560 nm. The DMSO is applied at the interface to match the refractive index. The color scale bar represents photon counts.

In Figure S3 we present the topography of the polymer bead used for the contact experiment depicted in Figure 2. This topography was subsequently employed to acquire the material's hardness, which was necessary for the BEM simulation. Figure S4 displays the emission spectra of molecule **1** under contact and at the free surface.

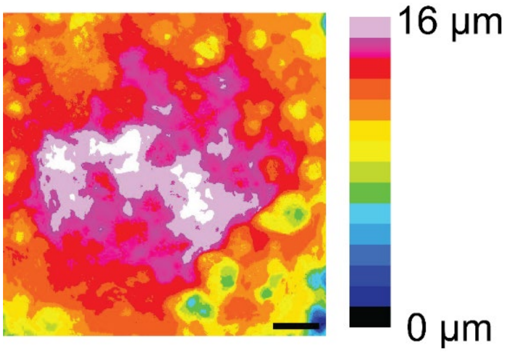

Figure S3. Topography image of the polymer bead used for the contact experiment in Figure 2.

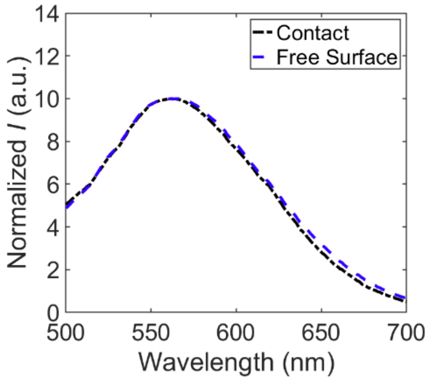

Figure S4 Emission spectra from immobilized molecule **1** at the free surface and at the contact interface, captured using the spectrometer on the microscope. The interface is wetted with DMSO to match the refractive index.

Figure S5 shows the simulated contact area based on the topography from Figure S3, adjusting the material's hardness (plasticity) between 50 MPa and 1000 Mpa. Comparing the experimental and simulated contact areas, we determined the hardness to be 200 Mpa. Table S2 lists the parameters for the contact simulation, sourced from a previous publication.[7]

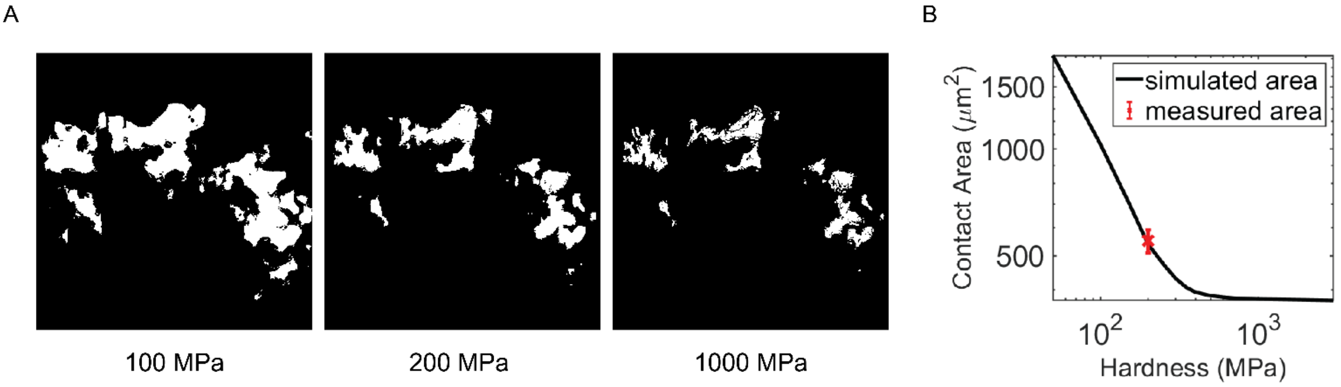

Figure S5 (A) Contact simulations for different hardness values. (B) Comparison of the simulated and the experimental contact areas, indicating that 200 Mpa is the hardness value that gives the best agreement. This parameter was further used in the BEM simulation for the experiment to determine the local pressure as shown in Figure 5.

Table S2. Input parameters for BEM simulation. *E* is the Young's modulus, ν the Poisson ratio, and *H* the plastic hardness of the material.

E_{glass} (Gpa)	E_{pmma} (Gpa)	ν_{glass}	ν_{PMMA}	H_{glass} (Mpa)	H_{pmma} (Mpa)
72	2.57	0.23	0.34	5500	200

In Figure S6, we display the topography of the polymer bead used for the contact experiment shown in Figure 5. Figure S7 presents the theoretical pressure image for a perfectly smooth bead with a radius of 1.45 mm. Using these pressure images, we plotted the distribution illustrated in Figure 5D.

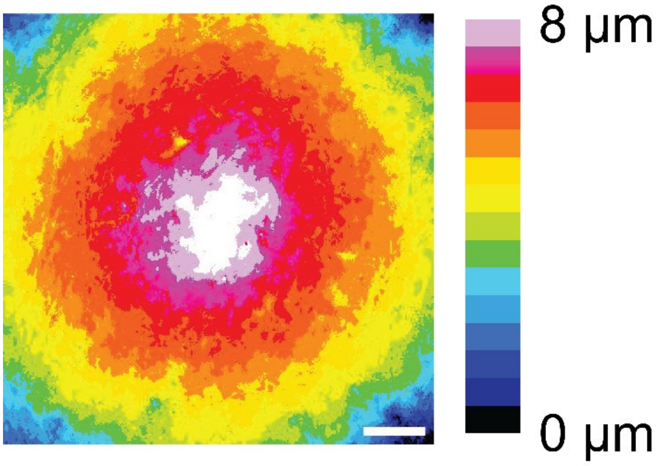

Figure S6. Topography of the polymer bead used for contact experiment in Figure 5. The scale bar represents 20 µm.

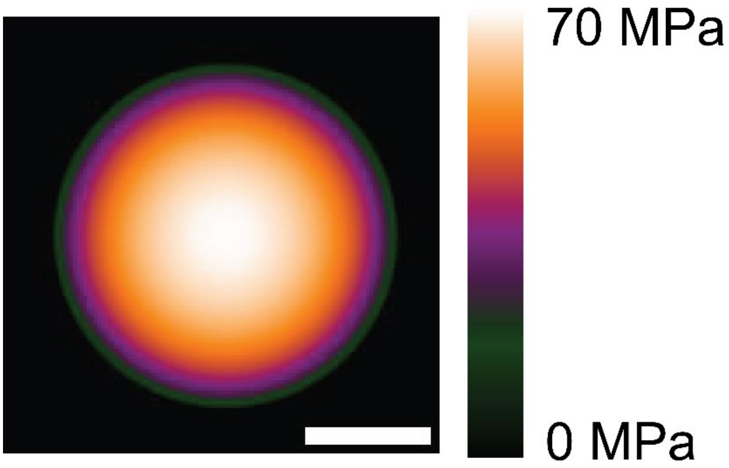

Figure S7. Theoretical pressure image of a perfectly smooth, i.e. single asperity, polymer bead with a radius of 1.45 mm. The scale bar represents 20 µm.

In Figure S8, we show the absorption and emission spectra of compounds **1** and **3** in THF. For hydrostatic pressure experiment, we present excitation and emission spectra of the model compound **3** in a toluene solution, serving as a stand-in for **1** which was too poorly soluble for this experiment. Molecule **3** displays a significant red shift in its spectra compared to molecule **1**. This is attributed to the stronger electron-withdrawing effect of the two cyano groups vs. a cyano and a COOH or CONR group, which further depresses the LUMO level of the molecule. Despite this difference, all three compounds can be expected to respond similarly to hydrostatic pressure. Details of the experimental setup can be found in the Materials and Methods section. Spectra were normalized based on peak intensity at 320 nm. The emission maxima shift by 12 nm (250 cm^{-1}) as pressure is increased from 0.1 Mpa to 270 Mpa. In the excitation spectra, we observed a red-shift of 14 nm (670 cm^{-1}) of the peak as pressure increased. Concurrently, the excitation intensity (or absorbance) also rises. However, this shift is not as pronounced as the effects observed at contact interfaces (Figure 2B): the intensity increased by 170% at 580 nm.

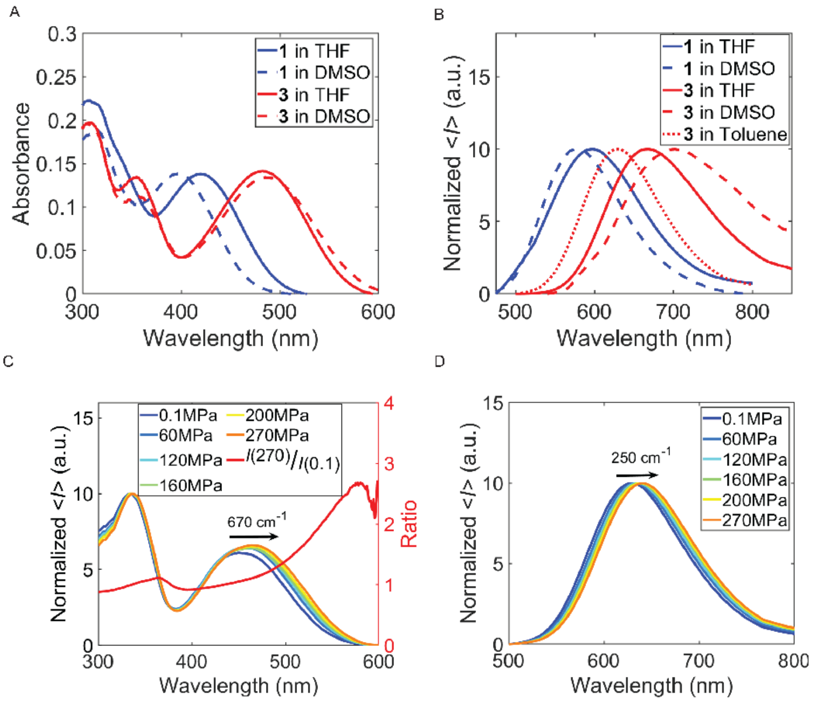

Figure S8 Absorption (A) and emission (B) spectra of **1** and **3** in THF and DMSO. Excitation (C) and emission (D) spectra of molecule **3** in toluene under hydrostatic pressure from 0.1 to 270 MPa.

In Figure S9 we show the relative single point energy (E_{rel}), the excitation energy (E_{exc}), and the oscillator strength (f) as a function of the dihedral angle ($\theta_{S-C-C-C}$) of the twisted molecules (cf. data in Table S1).

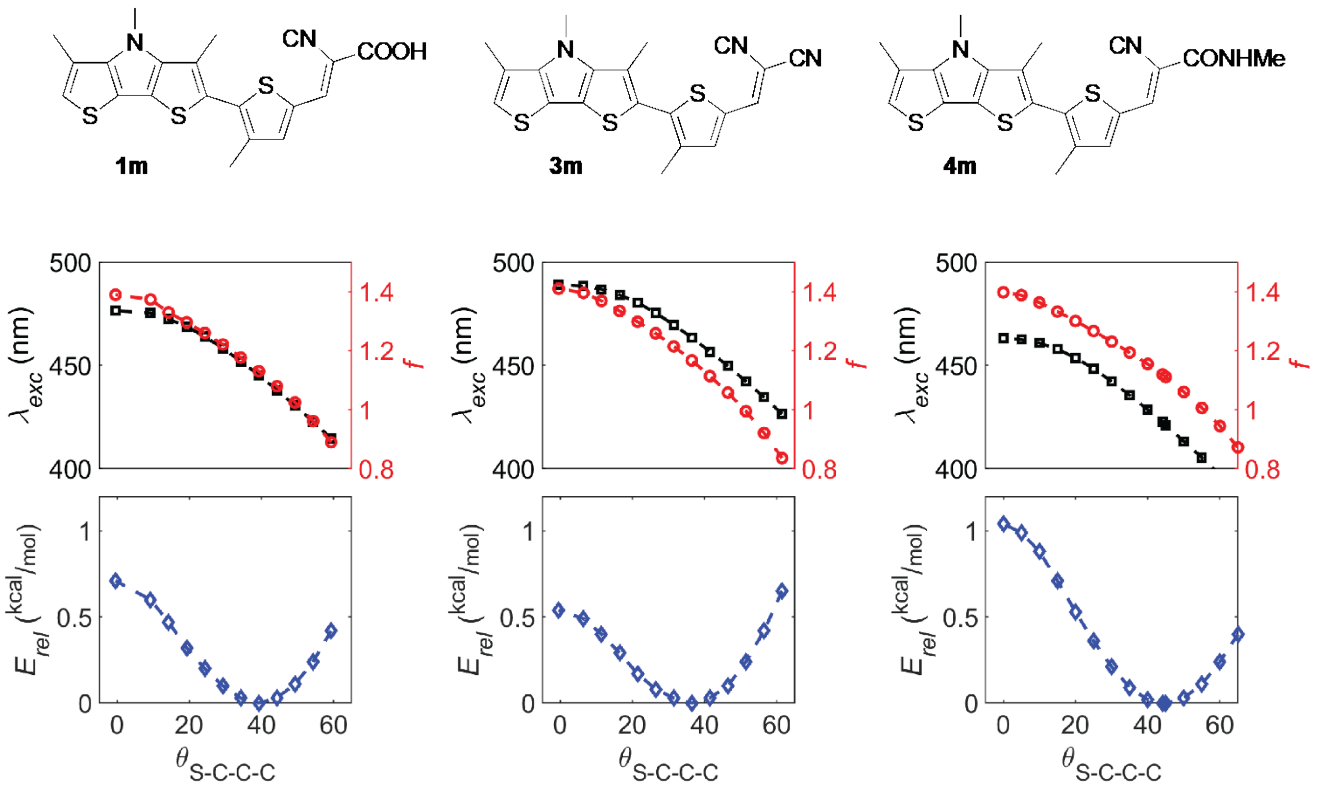

Figure S9. Computed values of the relative single point energy E_{rel}(PBE0/CC-pVTZ), excitation energy λ_{exc}, and oscillator strength f (CAM-B3LYP/CC-pVTZ) as a function of dihedral angle $\theta_{S-C-C-C}$ of the three model probes.

NMR, Infrared, and Mass Spectra

In Figures S10 to S24 we show ^{1}H, ^{13}C NMR, mass, and infrared spectra of **1**, **2**, **3**, and the intermediates.

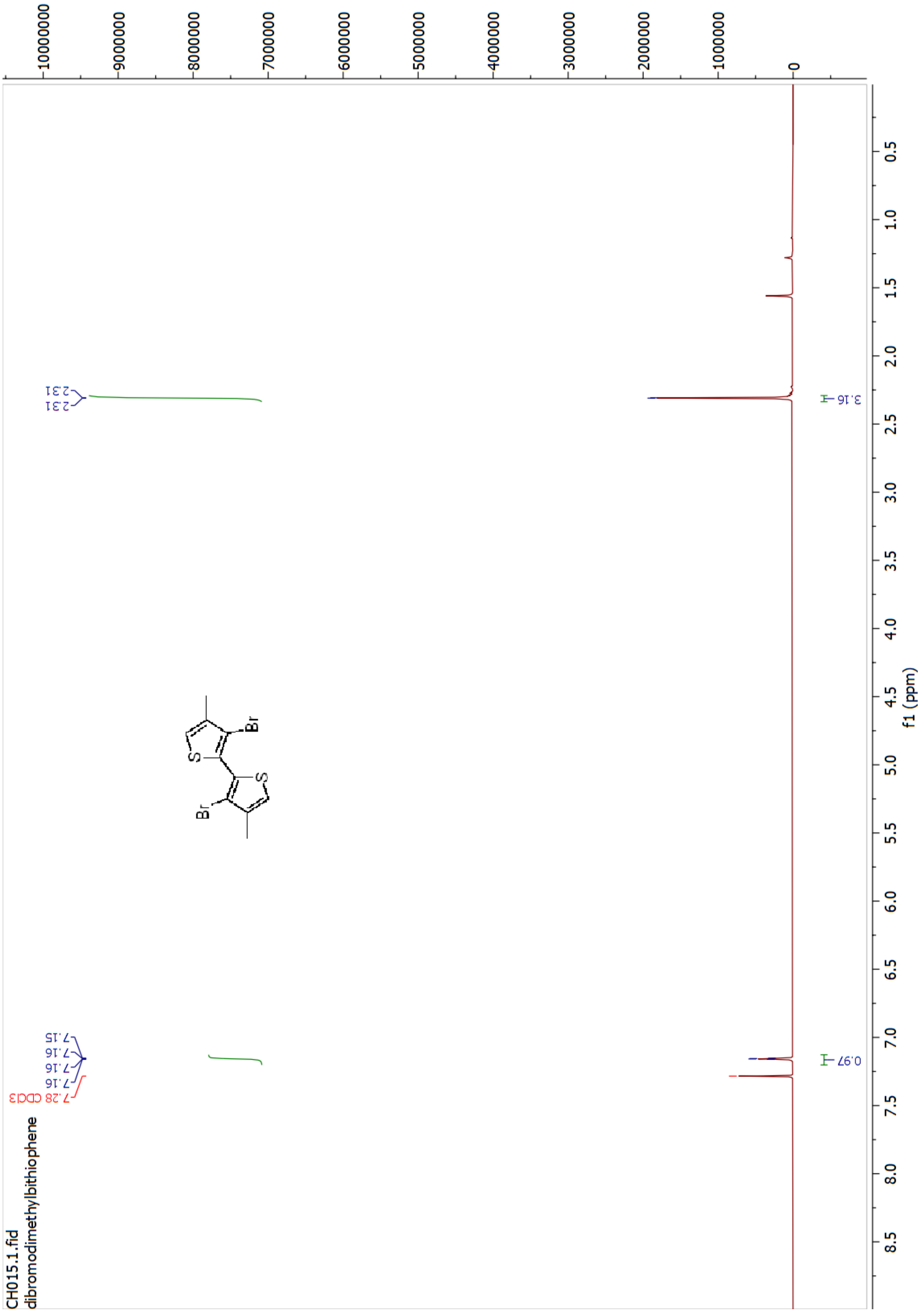

Figure S10 ^{1}H NMR spectrum of compound **c**. Solvent: $CDCl_3$.

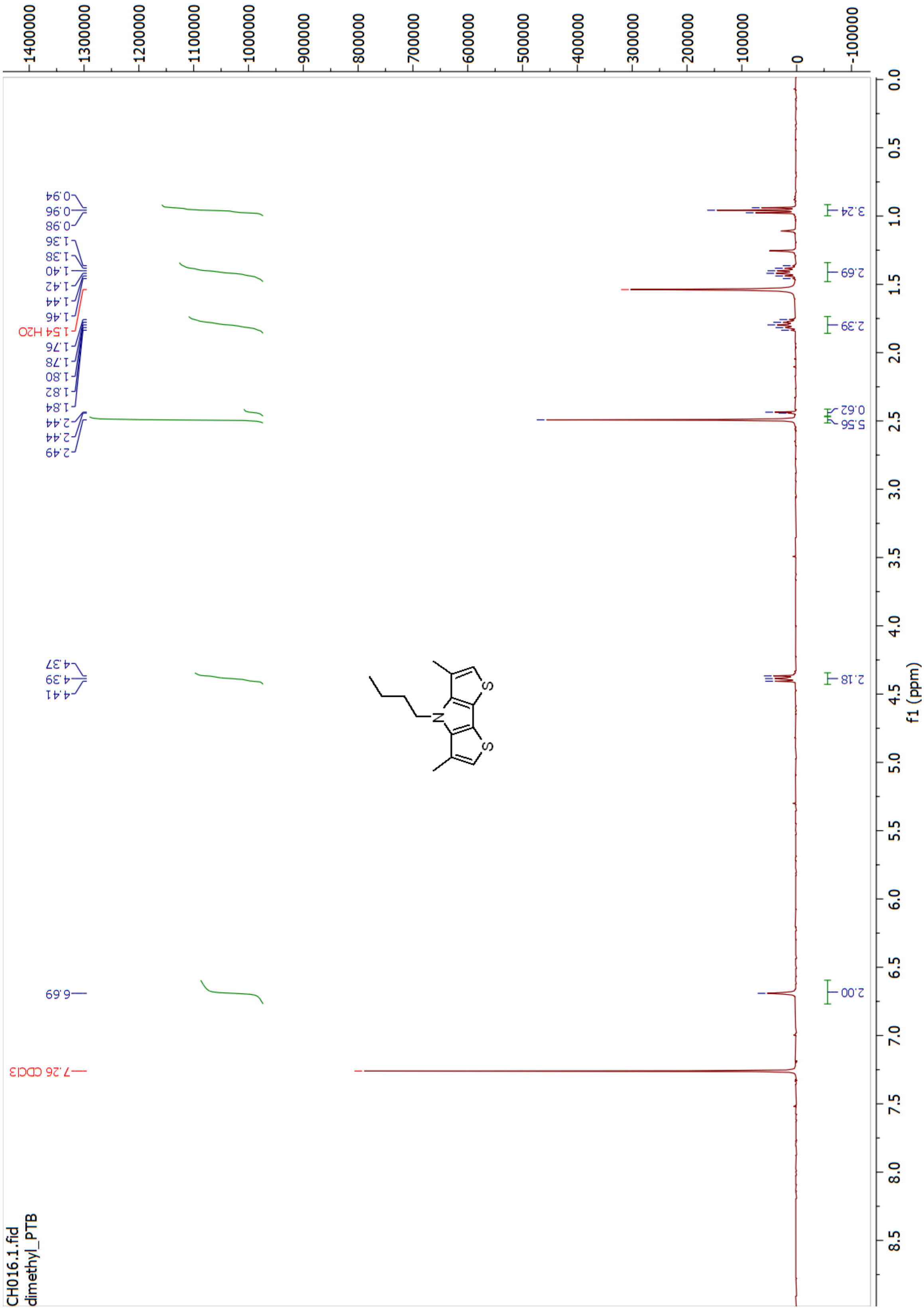

Figure S11 ^{1}H NMR spectrum of compound **d.** Solvent: $CDCl_3$

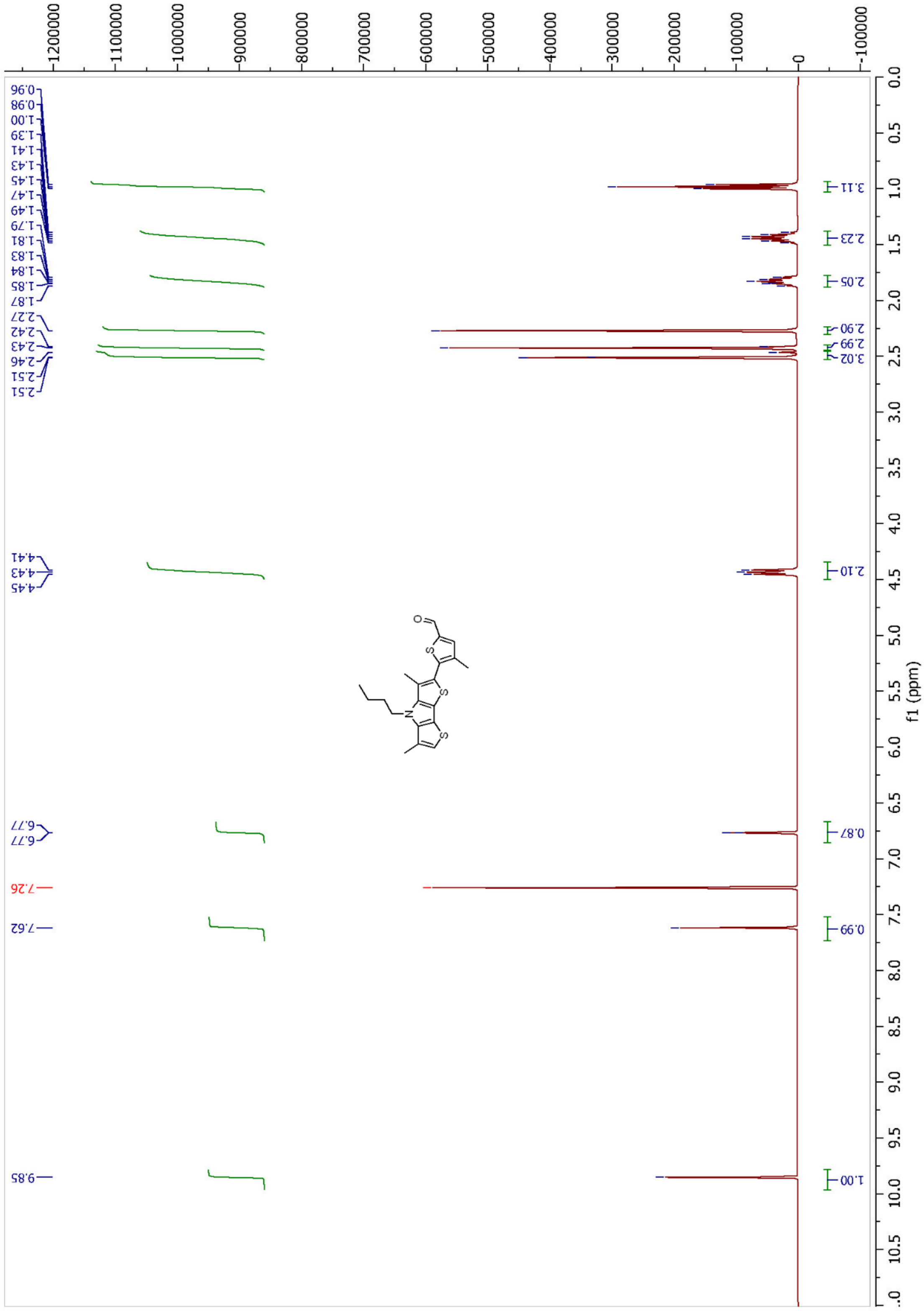

Figure S12 ^{1}H NMR spectrum of compound **e.** Solvent: $CDCl_3$.

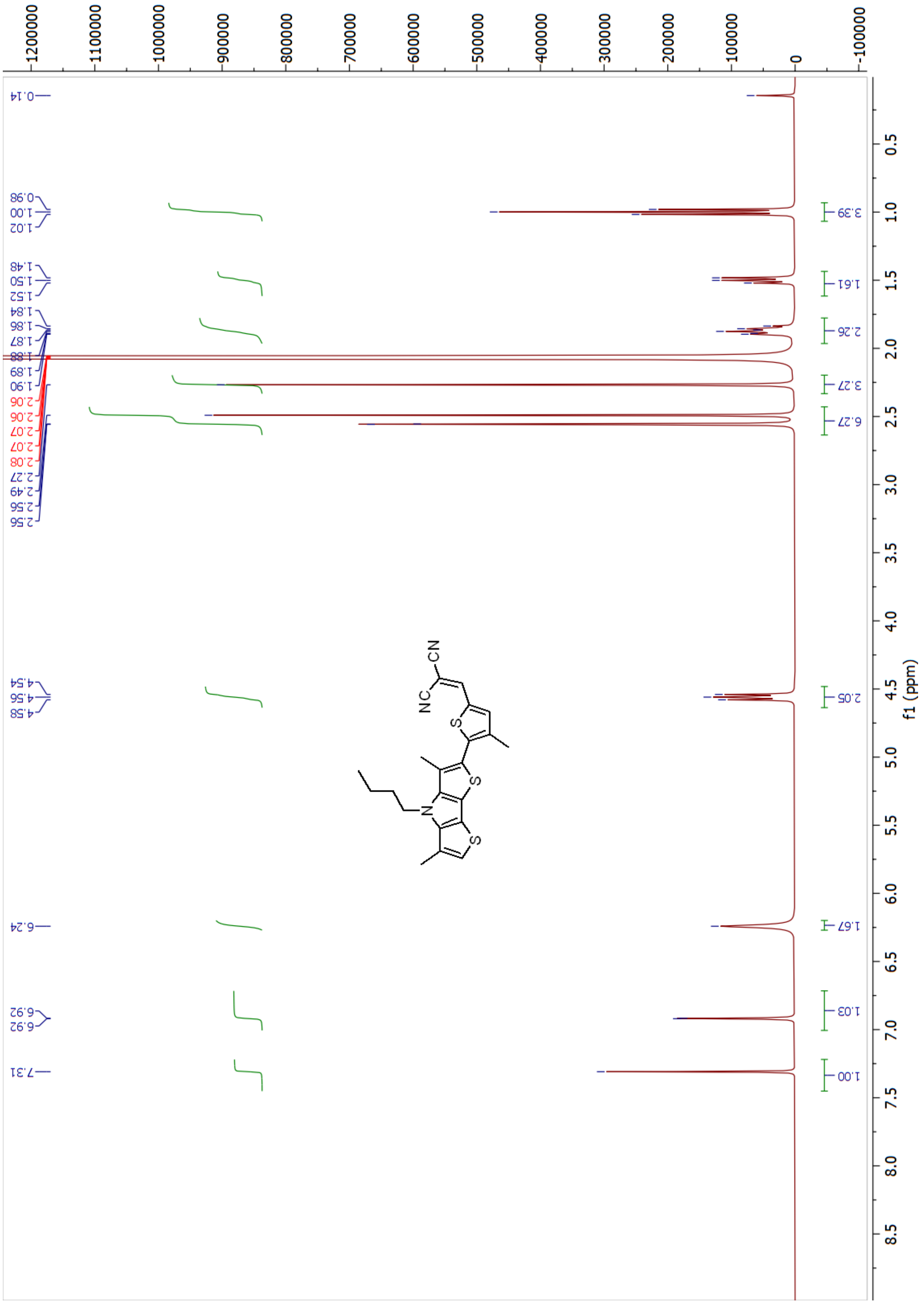

Figure S13 ^{1}H NMR spectrum of molecule **3**. Solvent: acetone-d$_6$.

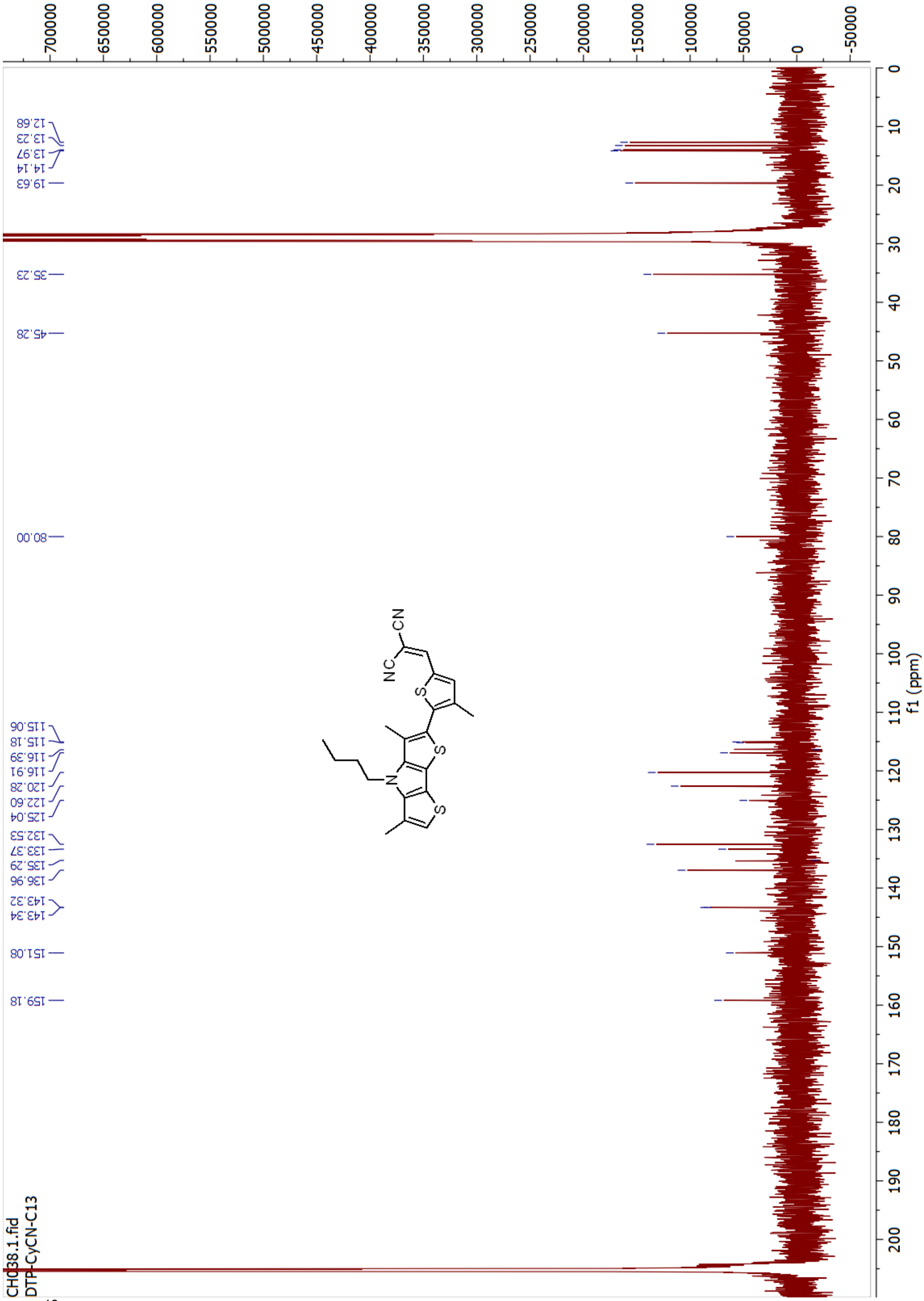

Figure S14 ^{13}C NMR spectrum of molecule **3**. Solvent: acetone-d$_6$.

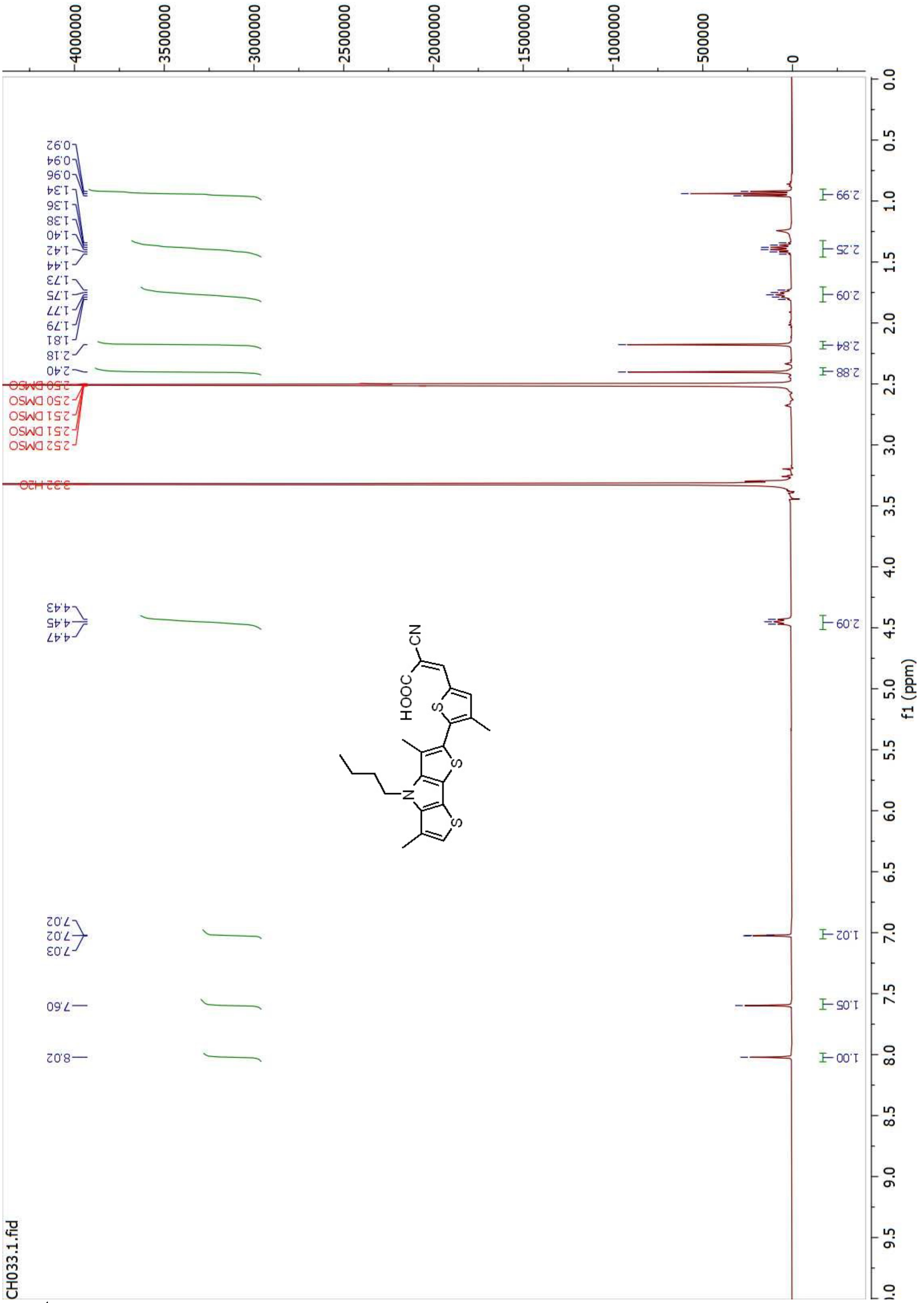

Figure S15 ^{1}H NMR spectrum of molecule **1**. Solvent: dmso-d_6.

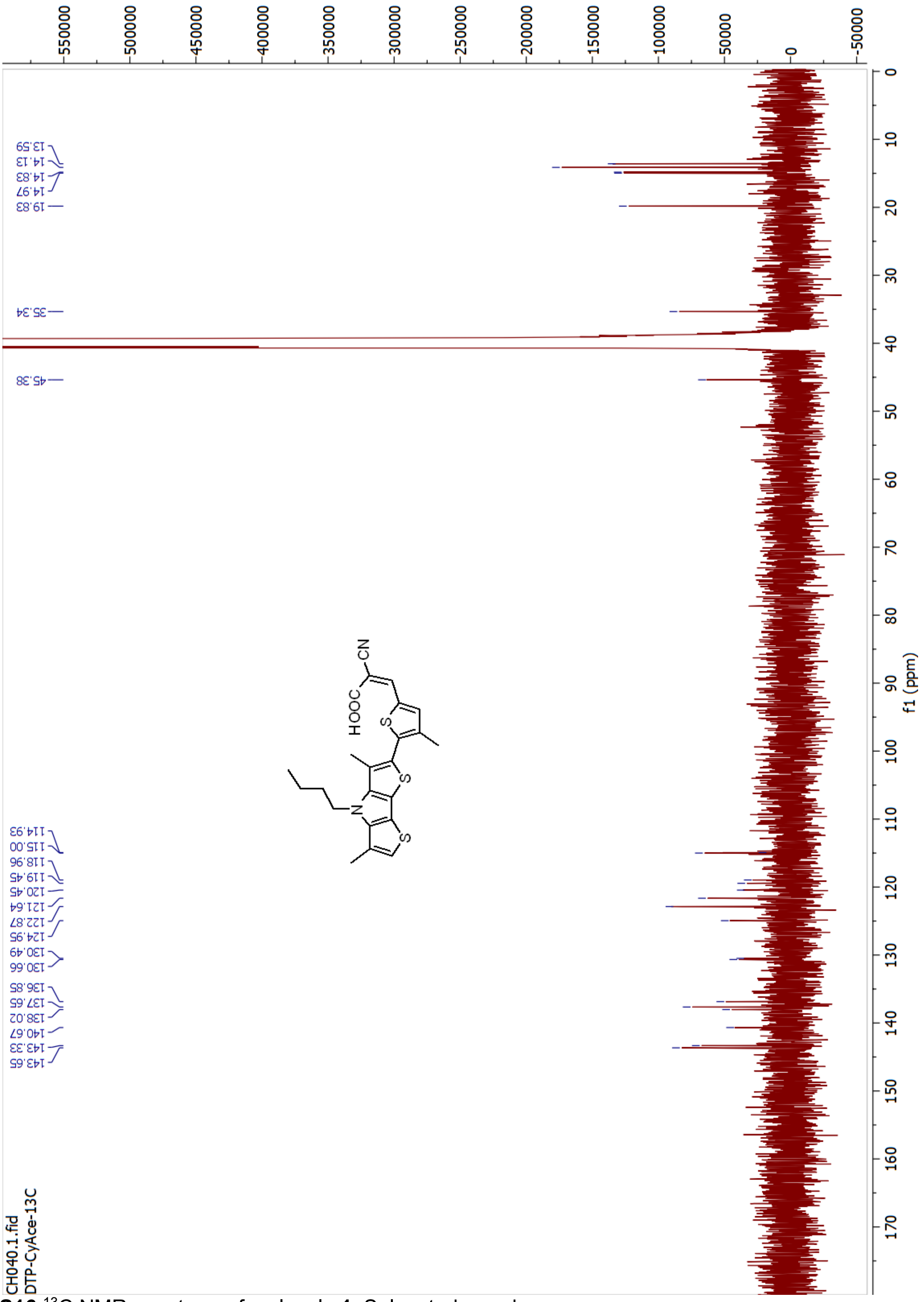

Figure S16 ^{13}C NMR spectrum of molecule **1**. Solvent: dmso-d_6.

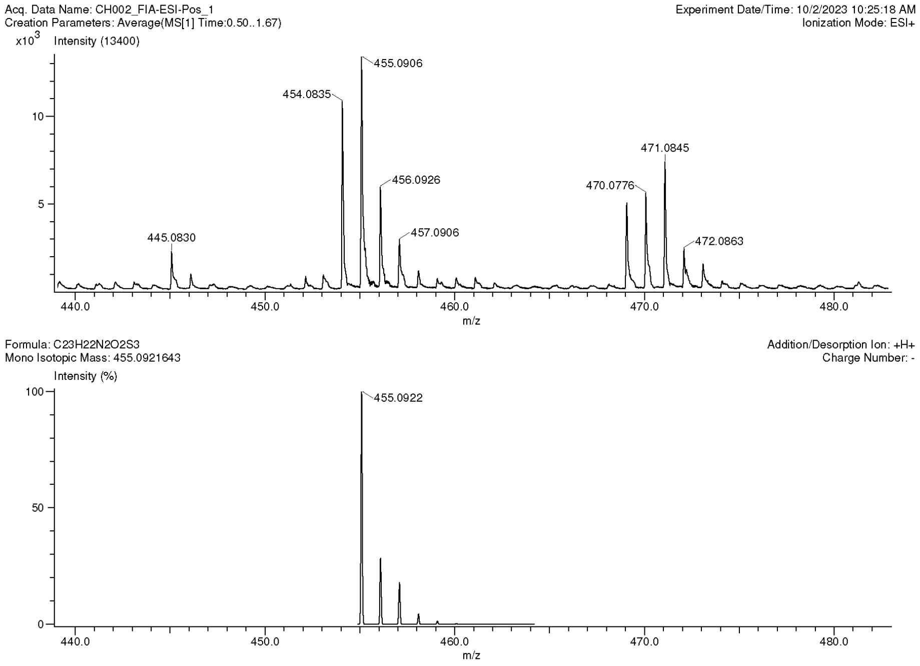

Figure S17 Mass spectrum of compound **1** Electrospray ionization method. Upper: measured. Lower: predicted.

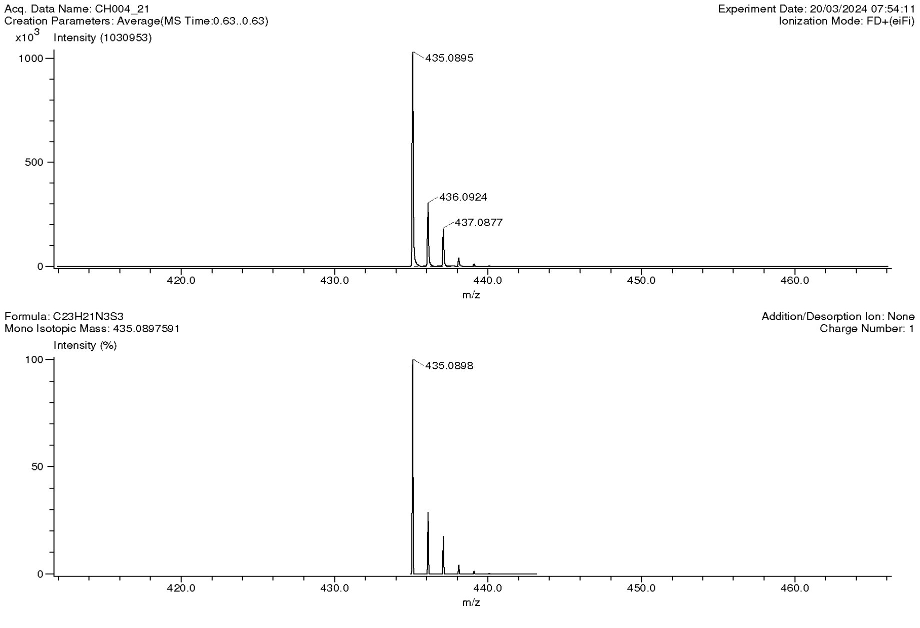

Figure S18 Mass spectrum of compound **3** using Field Desorption method. Upper: measured Lower: predicted.

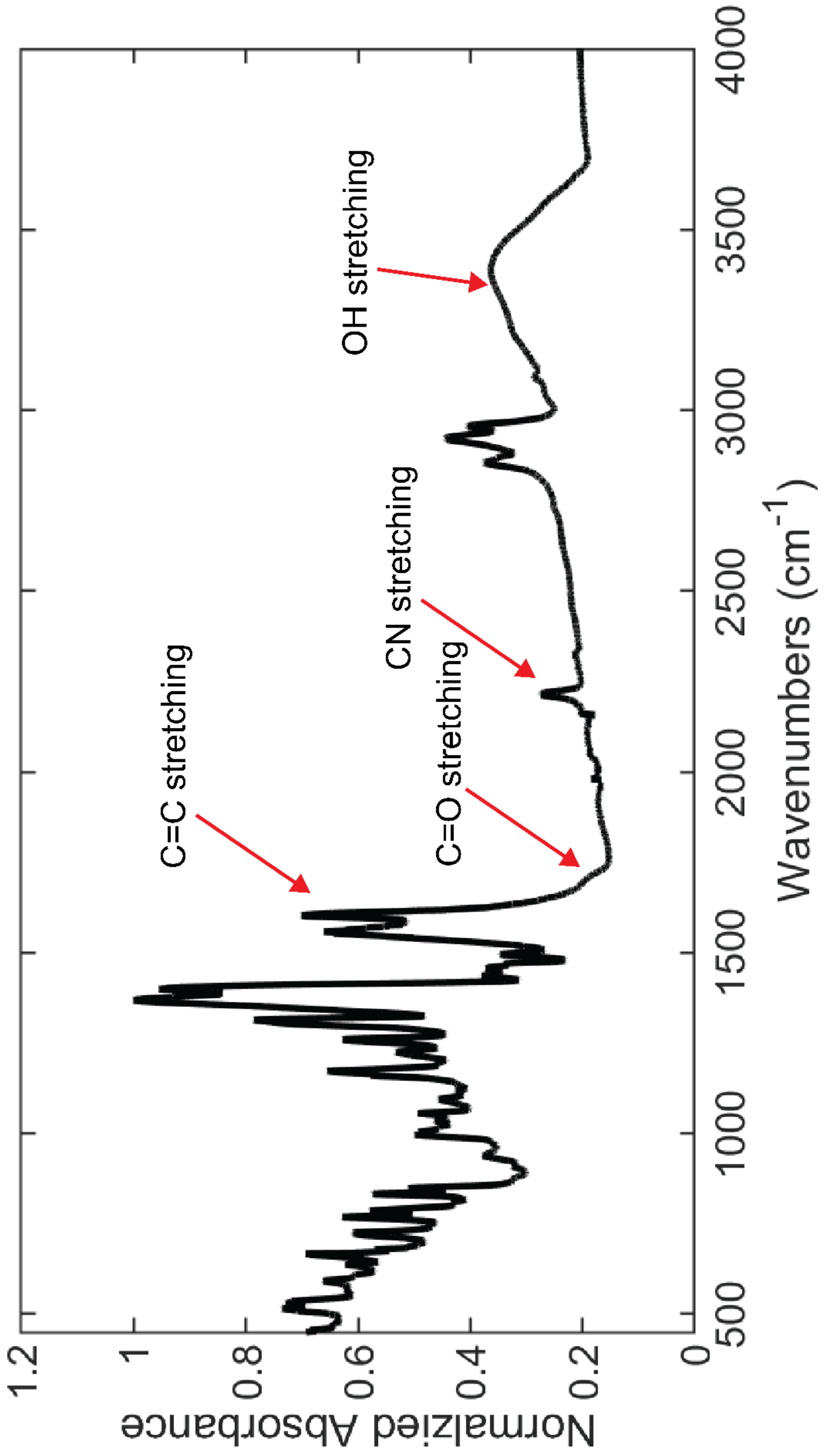

Figure S19 Infrared spectrum of compound **1** (solid powder).

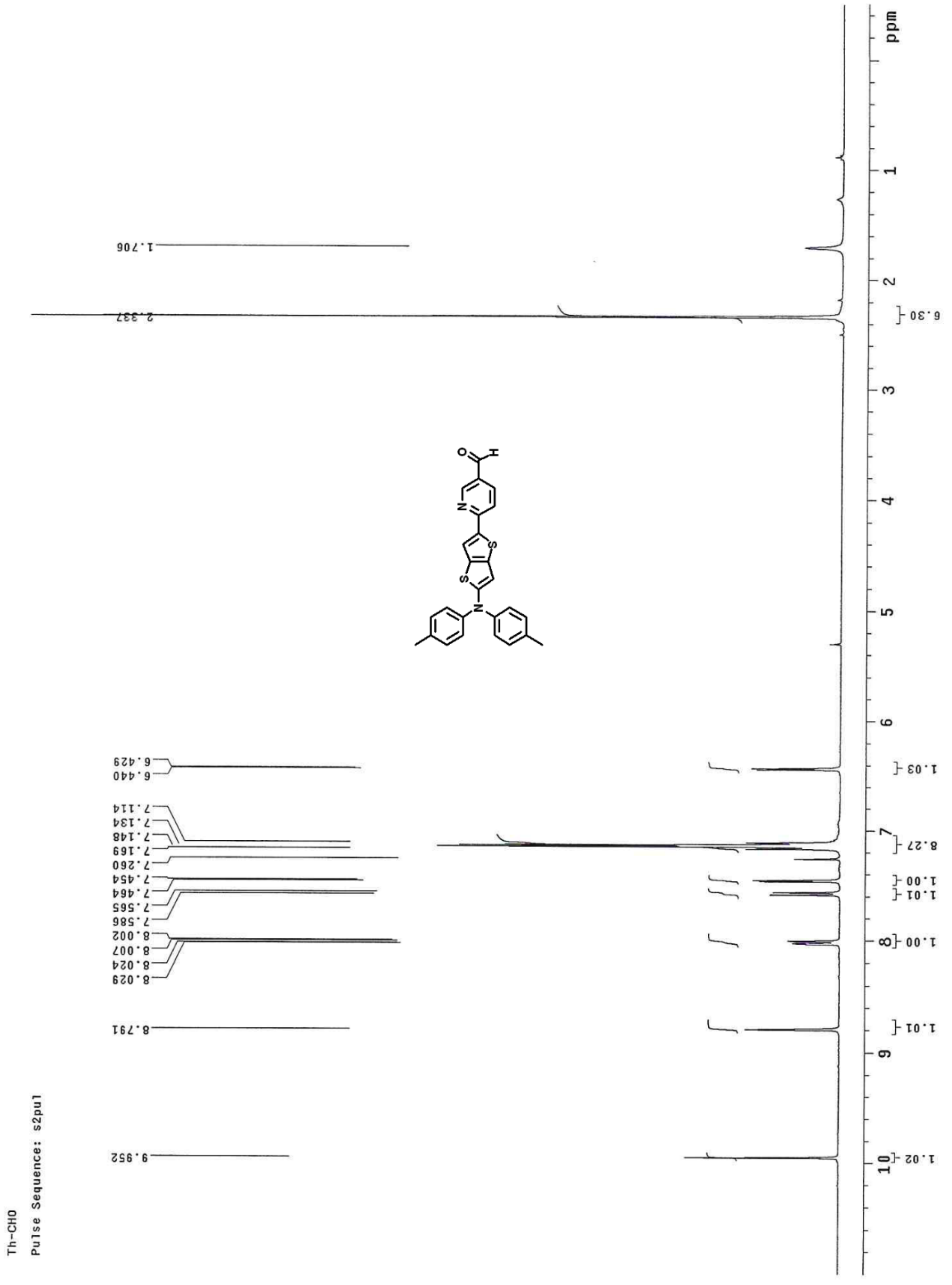

Figure S20 ^{1}H NMR spectrum of 6-(5-(di-p-tolylamino)thieno[3,2-b]thiophen-2-yl)nicotinaldehyde. Solvent: CD_2Cl_2.

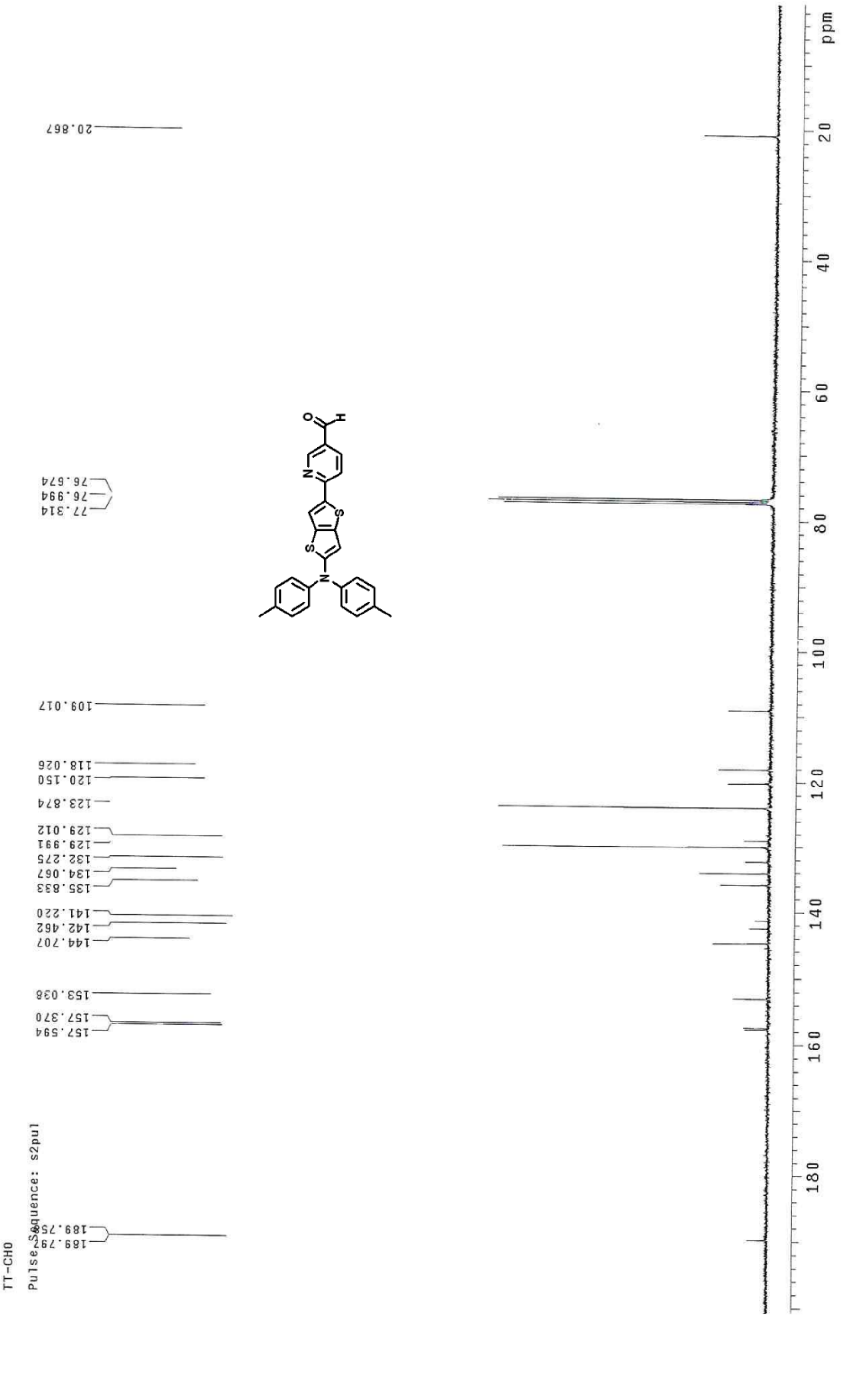

Figure S21 ^{13}C NMR spectrum of 6-(5-(di-p-tolylamino)thieno[3,2-b]thiophen-2-yl)nicotinaldehyde. Solvent: CD_2Cl_2.

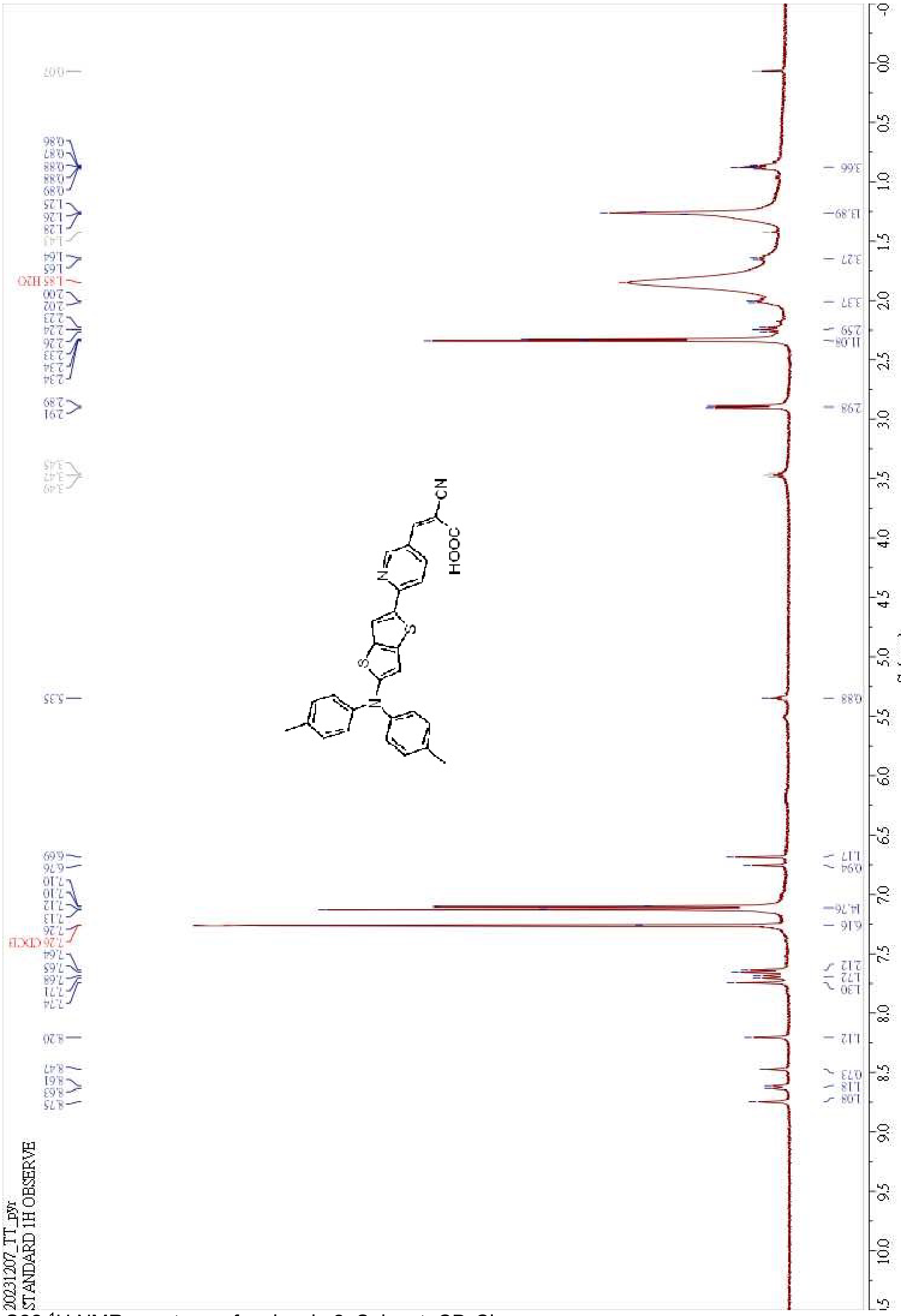

Figure S22 ^{1}H NMR spectrum of molecule **2**. Solvent: CD_2Cl_2.

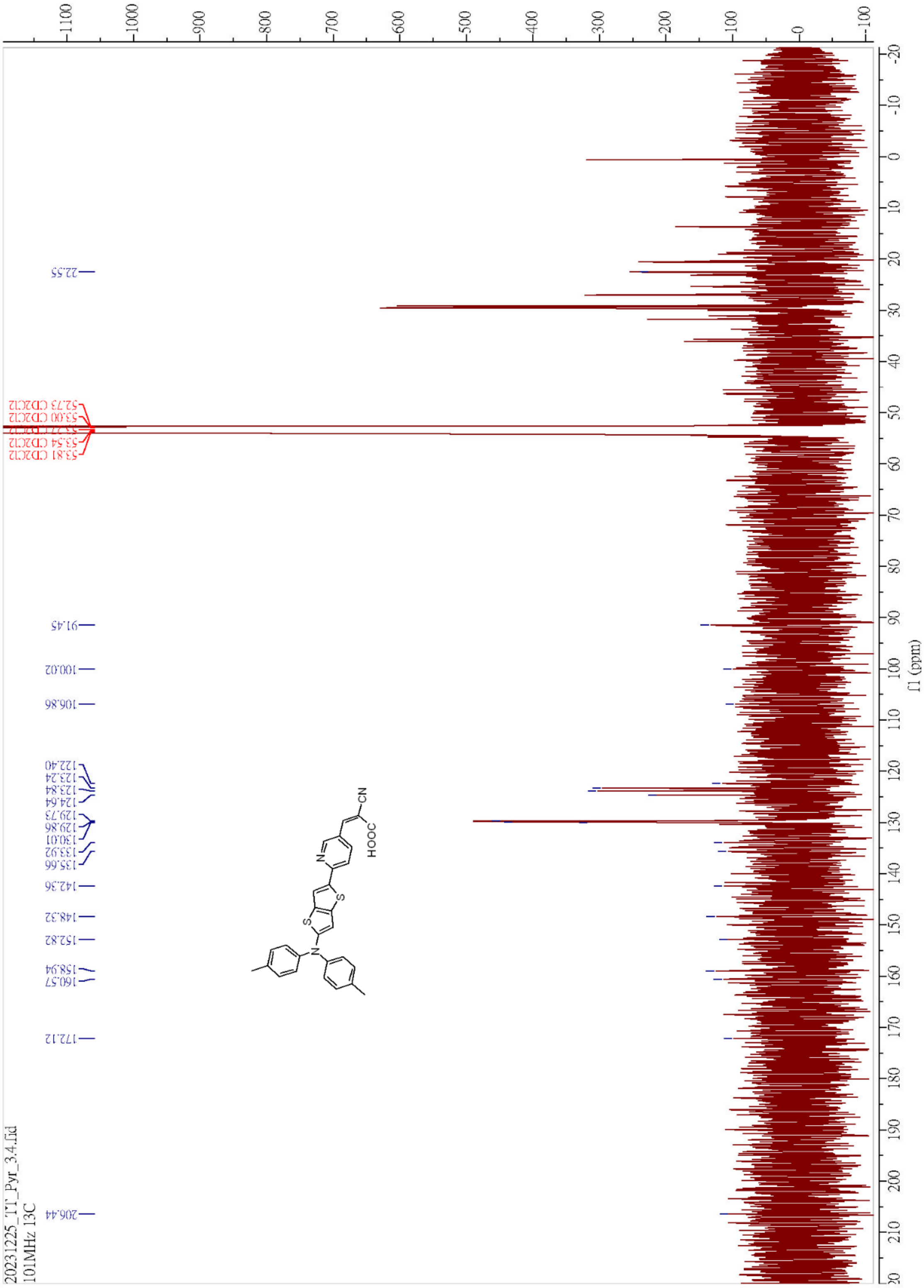

Figure S23 ^{13}C NMR spectrum of molecule **2**. Solvent: CD_2Cl_2.

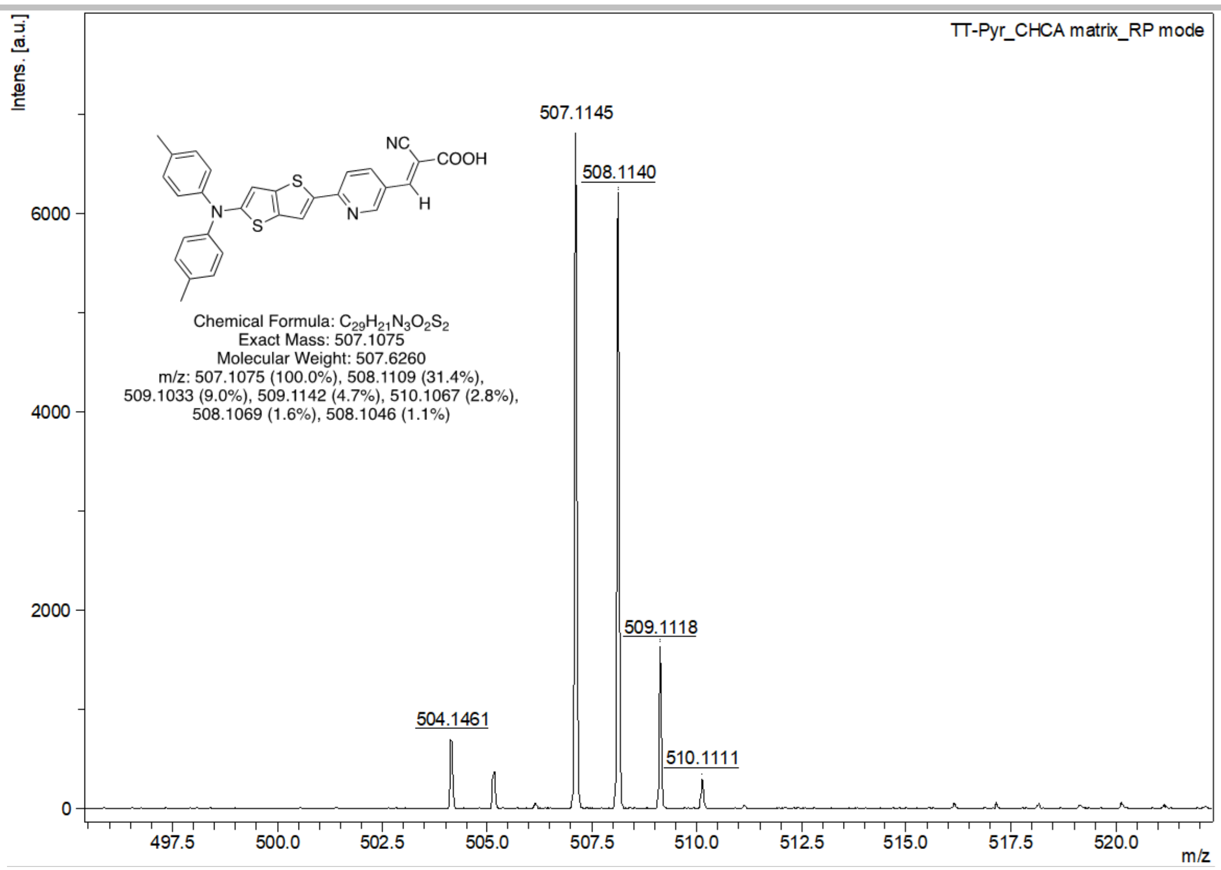

Figure S24 Mass spectrum of compound **2**.

References

(1) Mitsudo, K.; Shimohara, S.; Mizoguchi, J.; Mandai, H.; Suga, S. Synthesis of Nitrogen-Bridged Terthiophenes by Tandem Buchwald–Hartwig Coupling and Their Properties. *Org. Lett.* **2012**, *14* (11), 2702–2705. https://doi.org/10.1021/ol300887t.
(2) Kawabata, K.; Takeguchi, M.; Goto, H. Optical Activity of Heteroaromatic Conjugated Polymer Films Prepared by Asymmetric Electrochemical Polymerization in Cholesteric Liquid Crystals: Structural Function for Chiral Induction. *Macromolecules* **2013**, *46* (6), 2078–2091. https://doi.org/10.1021/ma400302j.
(3) Getmanenko, Y. A.; Tongwa, P.; Timofeeva, T. V.; Marder, S. R. Base-Catalyzed Halogen Dance Reaction and Oxidative Coupling Sequence as a Convenient Method for the Preparation of Dihalo-Bisheteroarenes. *Org. Lett.* **2010**, *12* (9), 2136–2139. https://doi.org/10.1021/ol1006423.
(4) Chen, C.-H.; Ting, H.-C.; Li, Y.-Z.; Lo, Y.-C.; Sher, P.-H.; Wang, J.-K.; Chiu, T.-L.; Lin, C.-F.; Hsu, I.-S.; Lee, J.-H.; Liu, S.-W.; Wong, K.-T. New D–A–A-Configured Small-Molecule Donors for High-Efficiency Vacuum-Processed Organic Photovoltaics under Ambient Light. *ACS Appl. Mater. Interfaces* **2019**, *11* (8), 8337–8349. https://doi.org/10.1021/acsami.8b20415.
(5) Chen, Y.-H.; Lin, L.-Y.; Lu, C.-W.; Lin, F.; Huang, Z.-Y.; Lin, H.-W.; Wang, P.-H.; Liu, Y.-H.; Wong, K.-T.; Wen, J.; Miller, D. J.; Darling, S. B. Vacuum-Deposited Small-Molecule Organic Solar Cells with High Power Conversion Efficiencies by Judicious Molecular Design and Device Optimization. *J. Am. Chem. Soc.* **2012**, *134* (33), 13616–13623. https://doi.org/10.1021/ja301872s.
(6) Suhina, T.; Weber, B.; Carpentier, C. E.; Lorincz, K.; Schall, P.; Bonn, D.; Brouwer, A. M. Fluorescence Microscopy Visualization of Contacts Between Objects. *Angew. Chem. Int. Ed.* **2015**, *54* (12), 3688–3691. https://doi.org/10.1002/anie.201410240.
(7) Hsu, C.-C.; Hsia, F.-C.; Weber, B.; De Rooij, M. B.; Bonn, D.; Brouwer, A. M. Local Shearing Force Measurement during Frictional Sliding Using Fluorogenic Mechanophores. *J. Phys. Chem. Lett.* **2022**, *13* (38), 8840–8844. https://doi.org/10.1021/acs.jpclett.2c02010.
(8) Abràmoff, M. D.; Magalhães, P. J.; Ram, S. J. Image Processing with imageJ. *Biophotonics International* **2004**, *11* (7), 36–41. https://doi.org/10.1201/9781420005615.ax4.
(9) Frisch, M. J.; Trucks, G. W.; Schlegel, H. B.; Scuseria, G. E.; Robb, M. A.; Cheeseman, J. R.; Scalmani, G.; Barone, V.; Petersson, G. A.; Nakatsuji, H.; Li, X.; Caricato, M.; Marenich, A. V.; Bloino, J.; Janesko, B. G.; Gomperts, R.; Mennucci, B.; Hratchian, H. P.; Ortiz, J. V.; Izmaylov, A. F.; Sonnenberg, J. L.; Williams; Ding, F.; Lipparini, F.; Egidi, F.; Goings, J.; Peng, B.; Petrone, A.; Henderson, T.; Ranasinghe, D.; Zakrzewski, V. G.; Gao, J.; Rega, N.; Zheng, G.; Liang, W.; Hada, M.; Ehara, M.; Toyota, K.; Fukuda, R.; Hasegawa, J.; Ishida, M.; Nakajima, T.; Honda, Y.; Kitao, O.; Nakai, H.; Vreven, T.; Throssell, K.; Montgomery Jr., J. A.; Peralta, J. E.; Ogliaro, F.; Bearpark, M. J.; Heyd, J. J.; Brothers, E. N.; Kudin, K. N.; Staroverov, V. N.; Keith, T. A.; Kobayashi, R.; Normand, J.; Raghavachari, K.; Rendell, A. P.; Burant, J. C.; Iyengar, S. S.; Tomasi, J.; Cossi, M.; Millam, J. M.; Klene, M.; Adamo, C.; Cammi, R.; Ochterski, J. W.; Martin, R. L.; Morokuma, K.; Farkas, O.; Foresman, J. B.; Fox, D. J. Gaussian 16 Rev. C.02, 2016.
(10) Caricato, M.; Mennucci, B.; Tomasi, J.; Ingrosso, F.; Cammi, R.; Corni, S.; Scalmani, G. Formation and Relaxation of Excited States in Solution: A New Time Dependent Polarizable Continuum Model Based on Time Dependent Density Functional Theory. *J. Chem. Phys.* **2006**, *124* (12), 124520. https://doi.org/10.1063/1.2183309.
(11) Epifanovsky, E.; Gilbert, A. T. B.; Feng, X.; Lee, J.; Mao, Y.; Mardirossian, N.; Pokhilko, P.; White, A. F.; Coons, M. P.; Dempwolff, A. L.; Gan, Z.; Hait, D.; Horn, P. R.; Jacobson, L. D.; Kaliman, I.; Kussmann, J.; Lange, A. W.; Lao, K. U.; Levine, D. S.; Liu, J.; McKenzie, S. C.;

Morrison, A. F.; Nanda, K. D.; Plasser, F.; Rehn, D. R.; Vidal, M. L.; You, Z.-Q.; Zhu, Y.; Alam, B.; Albrecht, B. J.; Aldossary, A.; Alguire, E.; Andersen, J. H.; Athavale, V.; Barton, D.; Begam, K.; Behn, A.; Bellonzi, N.; Bernard, Y. A.; Berquist, E. J.; Burton, H. G. A.; Carreras, A.; Carter-Fenk, K.; Chakraborty, R.; Chien, A. D.; Closser, K. D.; Cofer-Shabica, V.; Dasgupta, S.; de Wergifosse, M.; Deng, J.; Diedenhofen, M.; Do, H.; Ehlert, S.; Fang, P.-T.; Fatehi, S.; Feng, Q.; Friedhoff, T.; Gayvert, J.; Ge, Q.; Gidofalvi, G.; Goldey, M.; Gomes, J.; González-Espinoza, C. E.; Gulania, S.; Gunina, A. O.; Hanson-Heine, M. W. D.; Harbach, P. H. P.; Hauser, A.; Herbst, M. F.; Hernández Vera, M.; Hodecker, M.; Holden, Z. C.; Houck, S.; Huang, X.; Hui, K.; Huynh, B. C.; Ivanov, M.; Jász, Á.; Ji, H.; Jiang, H.; Kaduk, B.; Kähler, S.; Khistyaev, K.; Kim, J.; Kis, G.; Klunzinger, P.; Koczor-Benda, Z.; Koh, J. H.; Kosenkov, D.; Koulias, L.; Kowalczyk, T.; Krauter, C. M.; Kue, K.; Kunitsa, A.; Kus, T.; Ladjánszki, I.; Landau, A.; Lawler, K. V.; Lefrancois, D.; Lehtola, S.; Li, R. R.; Li, Y.-P.; Liang, J.; Liebenthal, M.; Lin, H.-H.; Lin, Y.-S.; Liu, F.; Liu, K.-Y.; Loipersberger, M.; Luenser, A.; Manjanath, A.; Manohar, P.; Mansoor, E.; Manzer, S. F.; Mao, S.-P.; Marenich, A. V.; Markovich, T.; Mason, S.; Maurer, S. A.; McLaughlin, P. F.; Menger, M. F. S. J.; Mewes, J.-M.; Mewes, S. A.; Morgante, P.; Mullinax, J. W.; Oosterbaan, K. J.; Paran, G.; Paul, A. C.; Paul, S. K.; Pavošević, F.; Pei, Z.; Prager, S.; Proynov, E. I.; Rák, Á.; Ramos-Cordoba, E.; Rana, B.; Rask, A. E.; Rettig, A.; Richard, R. M.; Rob, F.; Rossomme, E.; Scheele, T.; Scheurer, M.; Schneider, M.; Sergueev, N.; Sharada, S. M.; Skomorowski, W.; Small, D. W.; Stein, C. J.; Su, Y.-C.; Sundstrom, E. J.; Tao, Z.; Thirman, J.; Tornai, G. J.; Tsuchimochi, T.; Tubman, N. M.; Veccham, S. P.; Vydrov, O.; Wenzel, J.; Witte, J.; Yamada, A.; Yao, K.; Yeganeh, S.; Yost, S. R.; Zech, A.; Zhang, I. Y.; Zhang, X.; Zhang, Y.; Zuev, D.; Aspuru-Guzik, A.; Bell, A. T.; Besley, N. A.; Bravaya, K. B.; Brooks, B. R.; Casanova, D.; Chai, J.-D.; Coriani, S.; Cramer, C. J.; Cserey, G.; DePrince, A. E., III; DiStasio, R. A., Jr.; Dreuw, A.; Dunietz, B. D.; Furlani, T. R.; Goddard, W. A., III; Hammes-Schiffer, S.; Head-Gordon, T.; Hehre, W. J.; Hsu, C.-P.; Jagau, T.-C.; Jung, Y.; Klamt, A.; Kong, J.; Lambrecht, D. S.; Liang, W.; Mayhall, N. J.; McCurdy, C. W.; Neaton, J. B.; Ochsenfeld, C.; Parkhill, J. A.; Peverati, R.; Rassolov, V. A.; Shao, Y.; Slipchenko, L. V.; Stauch, T.; Steele, R. P.; Subotnik, J. E.; Thom, A. J. W.; Tkatchenko, A.; Truhlar, D. G.; Van Voorhis, T.; Wesolowski, T. A.; Whaley, K. B.; Woodcock, H. L., III; Zimmerman, P. M.; Faraji, S.; Gill, P. M. W.; Head-Gordon, M.; Herbert, J. M.; Krylov, A. I. Software for the Frontiers of Quantum Chemistry: An Overview of Developments in the Q-Chem 5 Package. *J. Chem. Phys.* **2021**, *155* (8), 084801. https://doi.org/10.1063/5.0055522.

(12) Scheurer, M.; Dreuw, A.; Epifanovsky, E.; Head-Gordon, M.; Stauch, T. Modeling Molecules under Pressure with Gaussian Potentials. *J. Chem. Theory Comput.* **2021**, *17* (1), 583–597. https://doi.org/10.1021/acs.jctc.0c01212.
